# Supplementary material for: Photothermal CO2 conversion to ethanol through photothermal heterojunction-nanosheet arrays
Source: Nat Commun. 2024 Jul 5;15:5639. doi: 10.1038/s41467-024-49928-0 (PMC11224241; doi:10.1038/s41467-024-49928-0)
Supplement: Supplementary file 1 — Supplementary Information [file 41467_2024_49928_MOESM1_ESM.pdf]

Supplementary Information

Photothermal CO_2 Conversion to Ethanol through Photothermal Heterojunction-Nanosheet Arrays

Xiaodong Li[1†], Li Li[2†], Xingyuan Chu[3], Xiaohui Liu[3], Guangbo Chen[3], Quanquan Guo[1], Zhen Zhang[4], Mingchao Wang[3], Shuming Wang[2], Alexander Tahn[5], Yongfu Sun[2]* and Xinliang Feng[1, 3]*

[1]Max Planck Institute of Microstructure Physics, Weinberg 2, Halle 06120, Germany.
[2]Hefei National Research Center for Physical Sciences at Microscale, University of Science and Technology of China, Hefei 230026, P. R. China.
[3]Faculty of Chemistry and Food Chemistry & Center for Advancing Electronics Dresden (cfaed), Dresden University of Technology, Dresden 01062, Germany.
[4]School of Chemistry and Materials Science, University of Science and Technology of China, Hefei 230026, P. R. China.
[5]Dresden Center for Nanoanalysis (DCN), Dresden University of Technology, Dresden 01069, Germany.
[†]These authors contributed equally: Xiaodong Li, Li Li. *Corresponding author. Email: yfsun@ustc.edu.cn, Xinliang.Feng@tu-dresden.de

Supplementary Figures

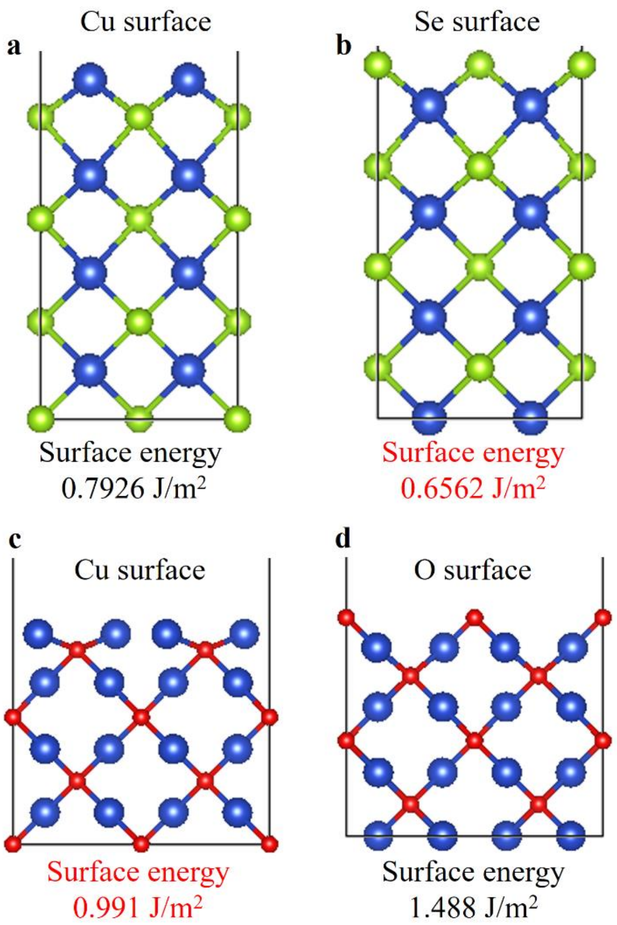

Supplementary Figure 1. The calculated surface energy with different exposed atoms. (a) Cu-surface Cu_2Se slab; (b) Se-surface Cu_2Se slab; (c) Cu-surface Cu_2O slab; (d) O-surface Cu_2O slab. From the above results, the Se-surface Cu_2Se and Cu-surface Cu_2O possess lower surface energy, indicating the more stable configuration, which are selected for the calculations of work function.

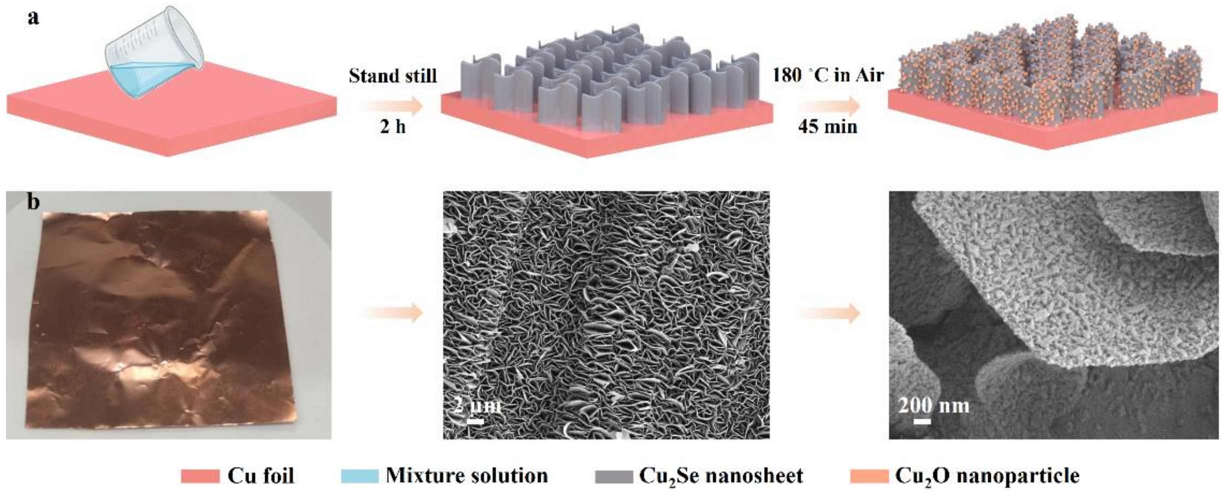

Supplementary Figure 2. Synthesis processes of CSCO heterojunction-nanosheet arrays (HNA) on Cu foil. (a) Schematic diagram; (b) digital and SEM images of the sample during different stages.

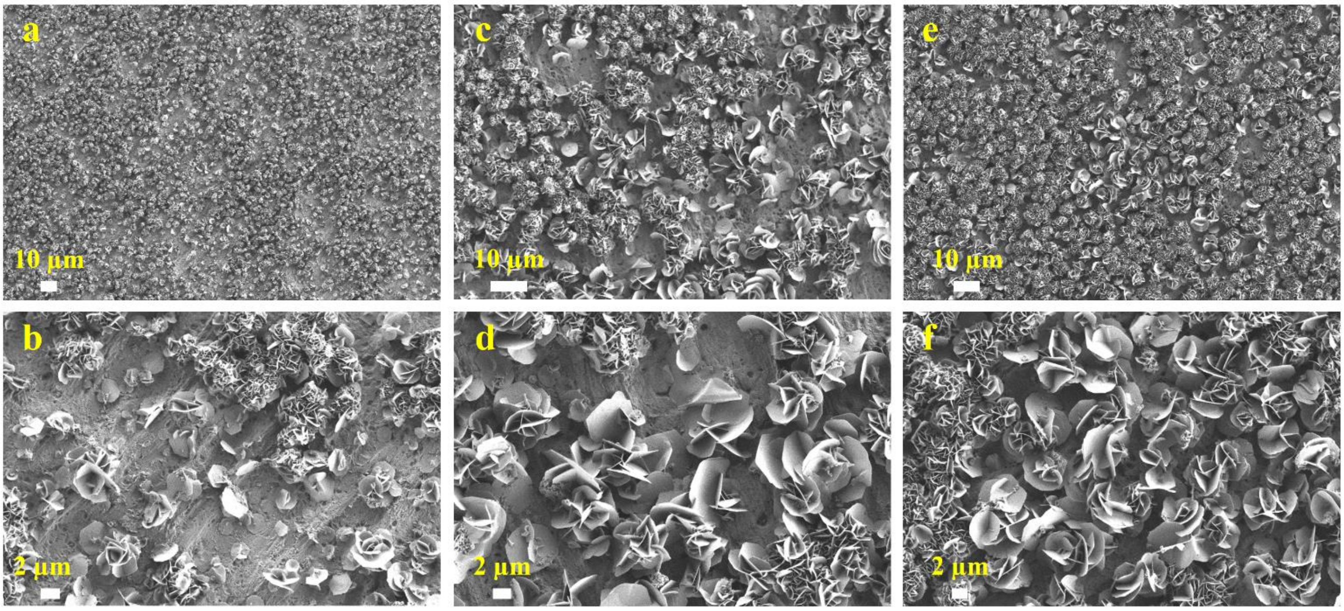

Supplementary Figure 3. The SEM images of L-Cu_2Se nanosheets on Cu foil with different reaction time. (a)-(b) 0.5 h; (c)-(d) 1 h; (e)-(f) 1.5 h.

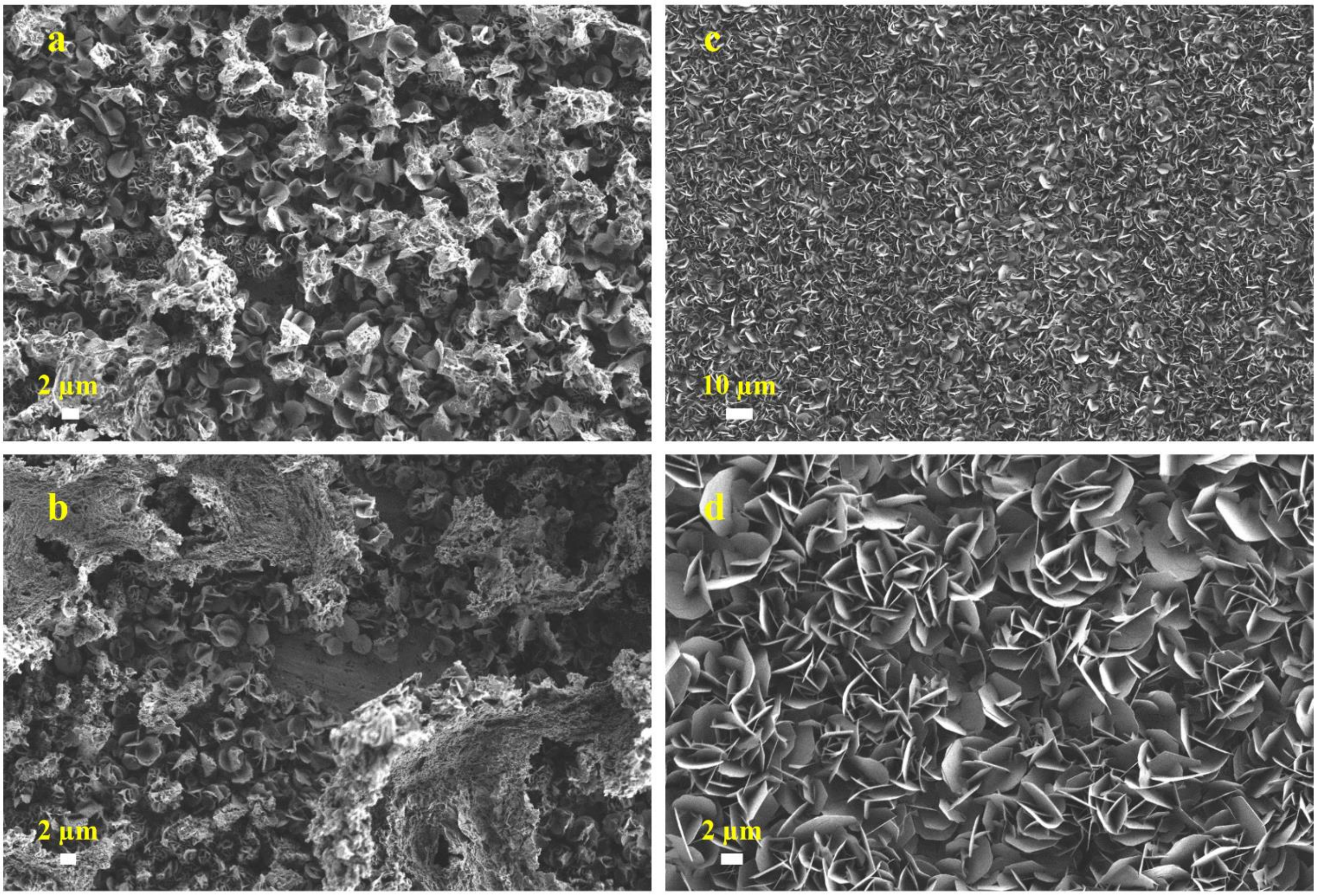

Supplementary Figure 4. The SEM images of L-Cu_2Se nanosheets on Cu foil with different concentration of Se precursor solution. (a)-(b) 0.5 times compared to the concentration used in the Method; (c)-(d) 2 times compared to the concentration used in the Method.

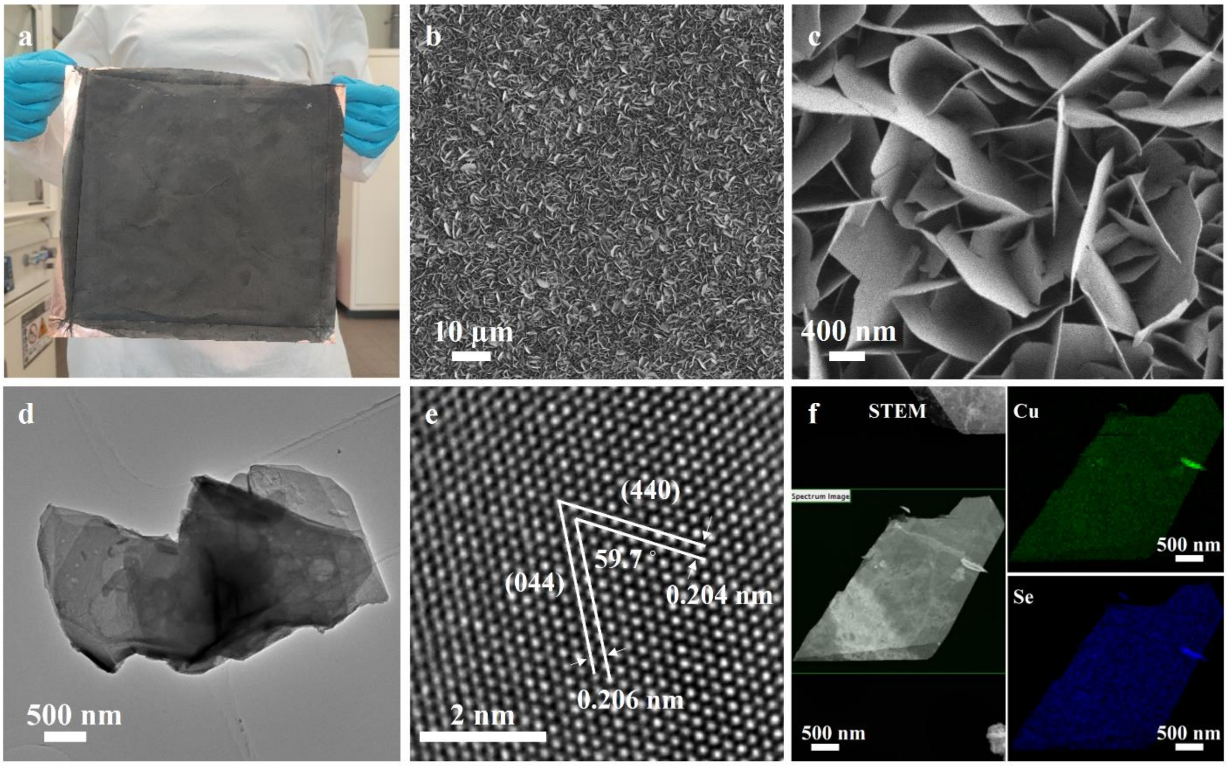

Supplementary Figure 5. Characterizations for L-Cu_2Se nanosheets. (a) Digital images of the large-scale Cu-Cu_2Se film; (b) and (c) SEM images at different scales; (d) TEM images; (e) HRTEM images; (f) STEM and EDS mapping images.

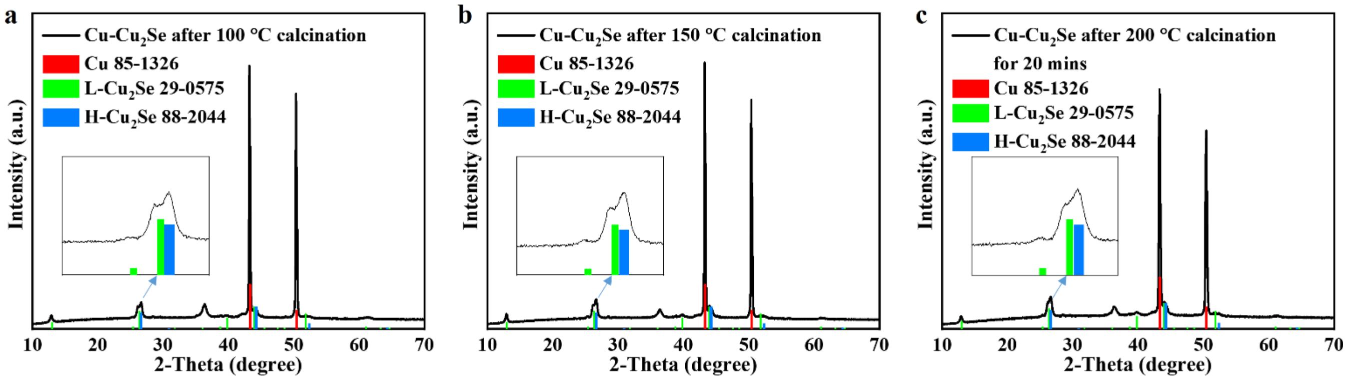

Supplementary Figure 6. XRD patterns of Cu-Cu_2Se under different conditions. (a) Cu-Cu_2Se after 100 ˚C for 45 mins. (b) Cu-Cu_2Se after 150 ˚C for 45 mins. (c) Cu-Cu_2Se after 200 ˚C for 20 mins.

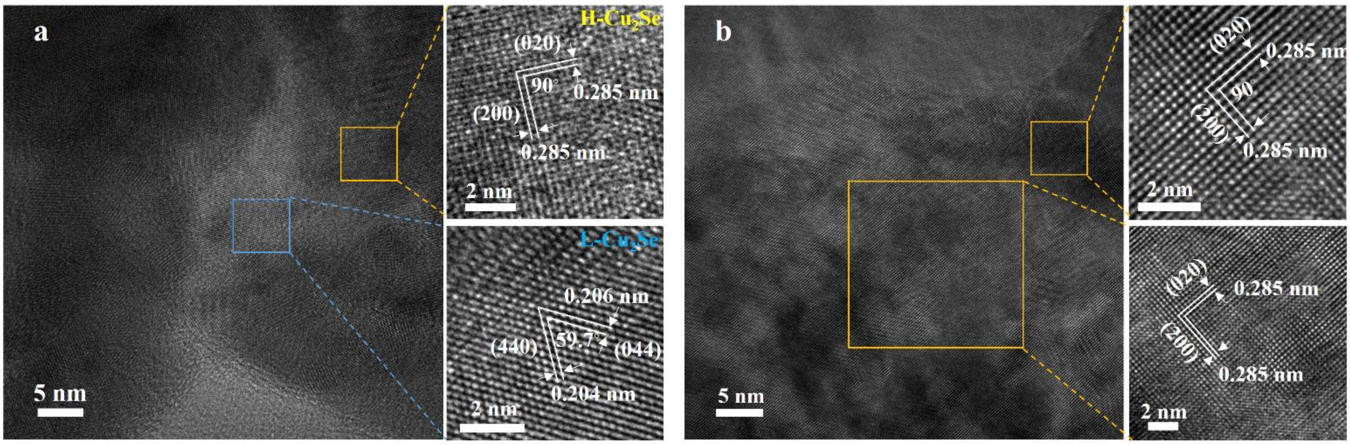

Supplementary Figure 7. HRTEM images during the conversion process. (a) Mixture phase of low-temperature-phase Cu_2Se (L-Cu_2Se) and high-temparature-phase Cu_2Se (H-Cu_2Se) after calcination for 20 minutes; (b) H-Cu_2Se after the total conversion process. Of note, after full calcination, the L-Cu_2Se was completely converted to H-Cu_2Se. As the result, a single phase of H-Cu_2Se obtained, which would be used for additional modifications.

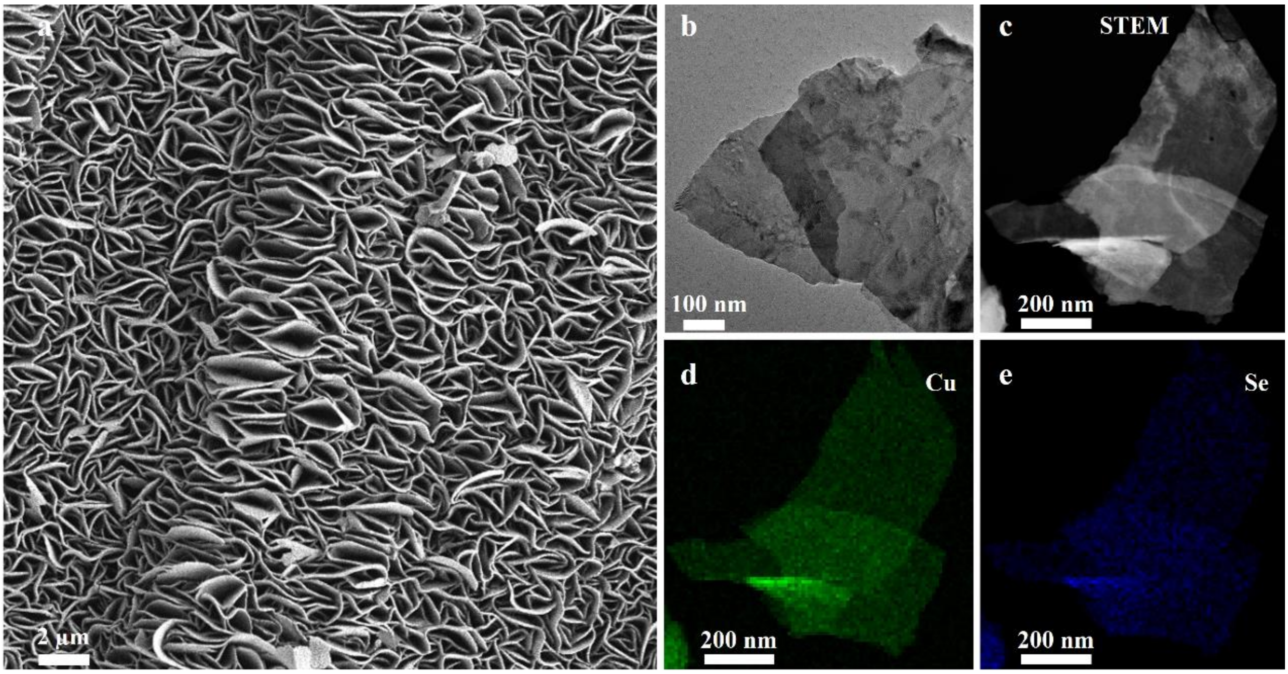

Supplementary Figure 8. Characterization for H-Cu_2Se nanosheets. (a) SEM images; (b) TEM images; (c)-(e) STEM and EDS mapping images.

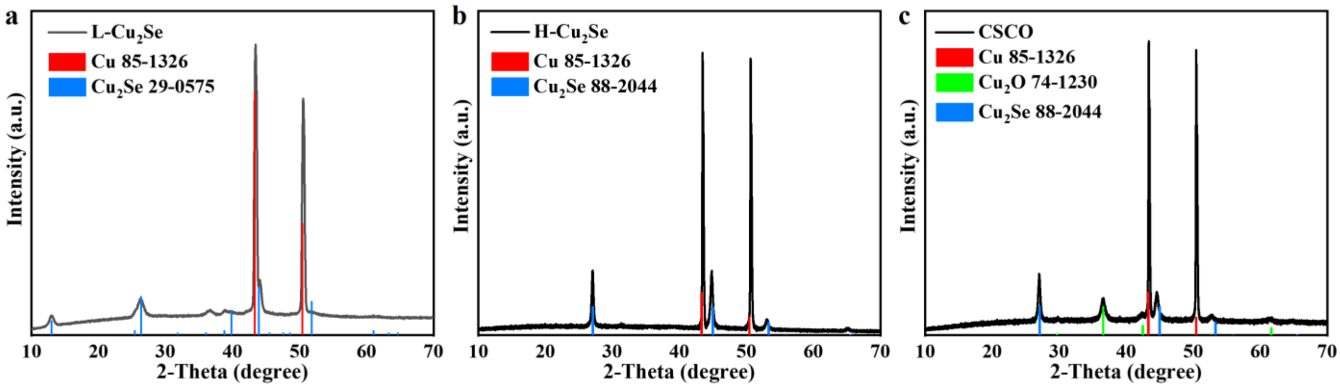

Supplementary Figure 9. XRD pattern. (a) L-Cu_2Se nanosheets on Cu foil, (b) H-Cu_2Se nanosheets on Cu foil and (c) Cu_2Se-Cu_2O (CSCO) HNA *in situ* grown on Cu foil.

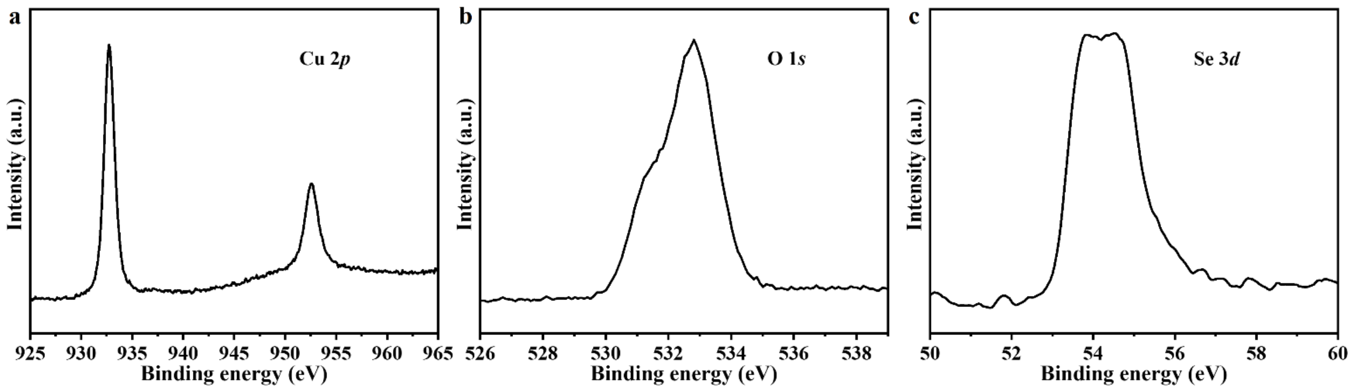

Supplementary Figure 10. XPS spectrum of CSCO HNA *in situ* grown on Cu foil. (a) High-resolution Cu 2*p* spectra; (b) high-resolution O 1*s* spectra; (c) high-resolution Se 3*d* spectra.

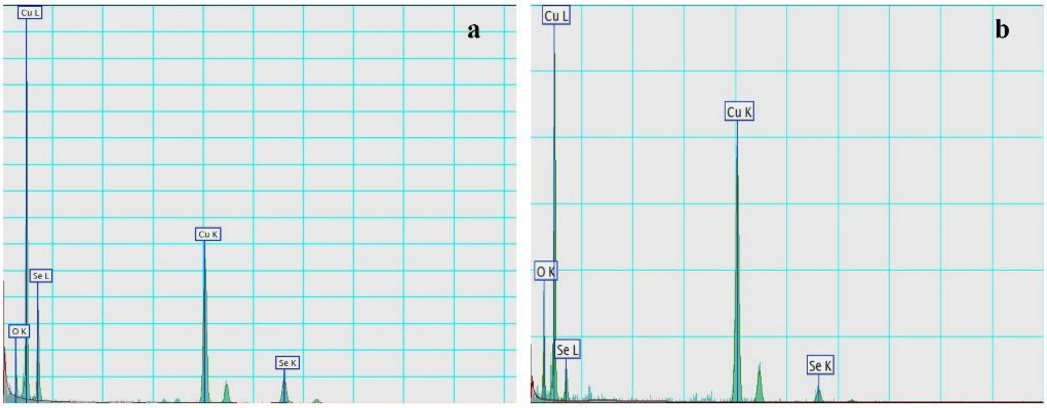

EDS element content of TEM							
	Element	Shell	Fitting Mode	Comp. (at.%)	k-Factor (/Si)	k-Factor Mode	Det. Corr.
Cu_2Se	**O**	**K**	**Theoretical**	**7.7**	**1.251**	**Theoretical**	**0.79**
	Se	K	Theoretical	17.0	2.655	Theoretical	1.0
	Cu	K	Theoretical	75.4	1.794	Theoretical	1.0
Cu_2Se-Cu_2O	**O**	**K**	**Theoretical**	**30.7**	**1.251**	**Theoretical**	**0.79**
	Se	K	Theoretical	5.31	2.655	Theoretical	1.0
	Cu	K	Theoretical	63.94	1.794	Theoretical	1.0

Supplementary Figure 11. TEM-extended EDS spectrum of (a) the H-Cu_2Se nanosheets and (b) CSCO heterojunction. The below is the element content analysis of H-Cu_2Se nanosheets and CSCO heterojunction. The corresponding samples for TEM and EDS mapping are obtained from the H-Cu_2Se nanosheets arrays and CSCO HNA *in situ* grown on Cu foil by sonication in ethanol.

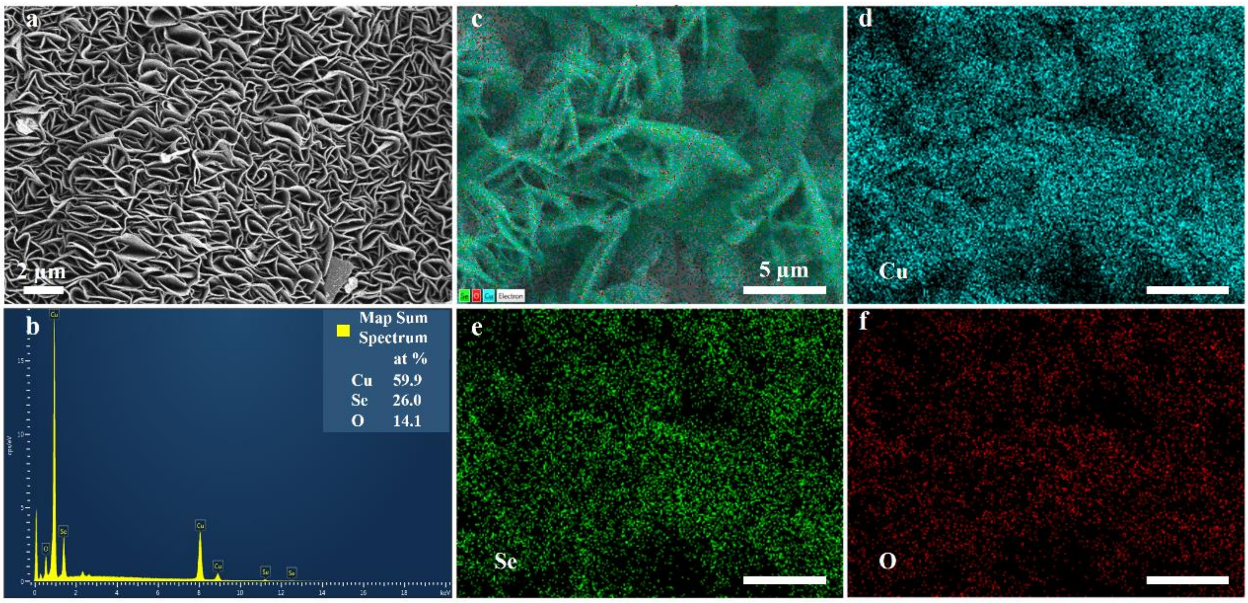

Supplementary Figure 12. Characterizations of CSCO HNA *in situ* grown on Cu bulk. (a) SEM image; (b) EDS spectrum and element content analysis; (c)-(f) element mapping images.

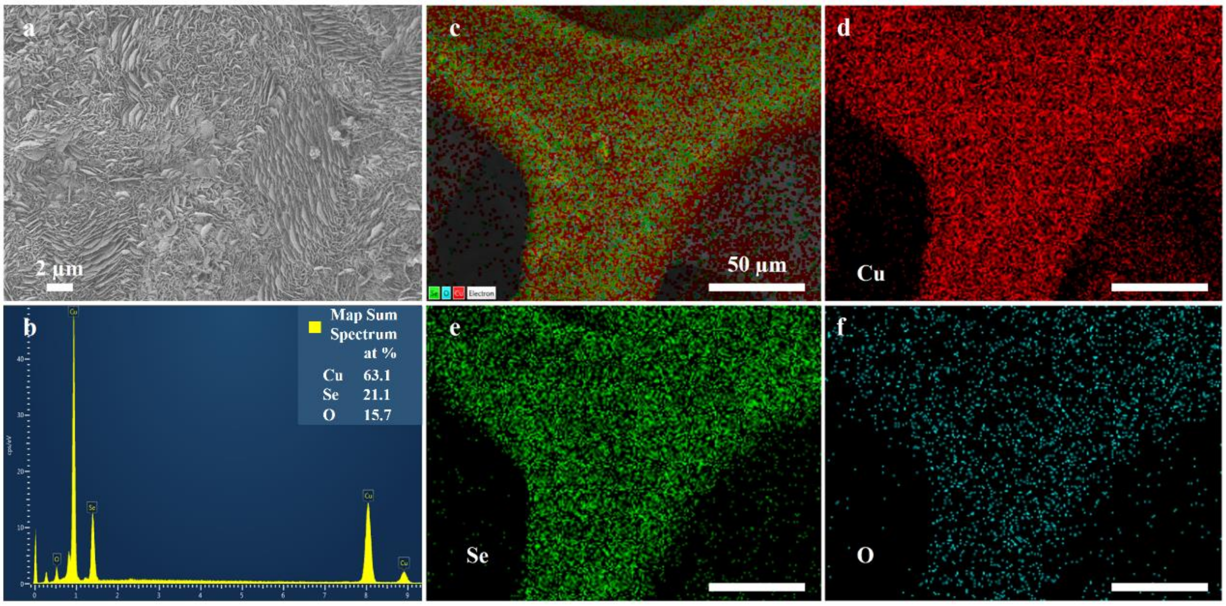

Supplementary Figure 13. Characterizations of CSCO HNA *in situ* grown on Cu foam. (a) SEM image; (b) EDS spectrum and element content analysis; (c)-(f) element mapping images.

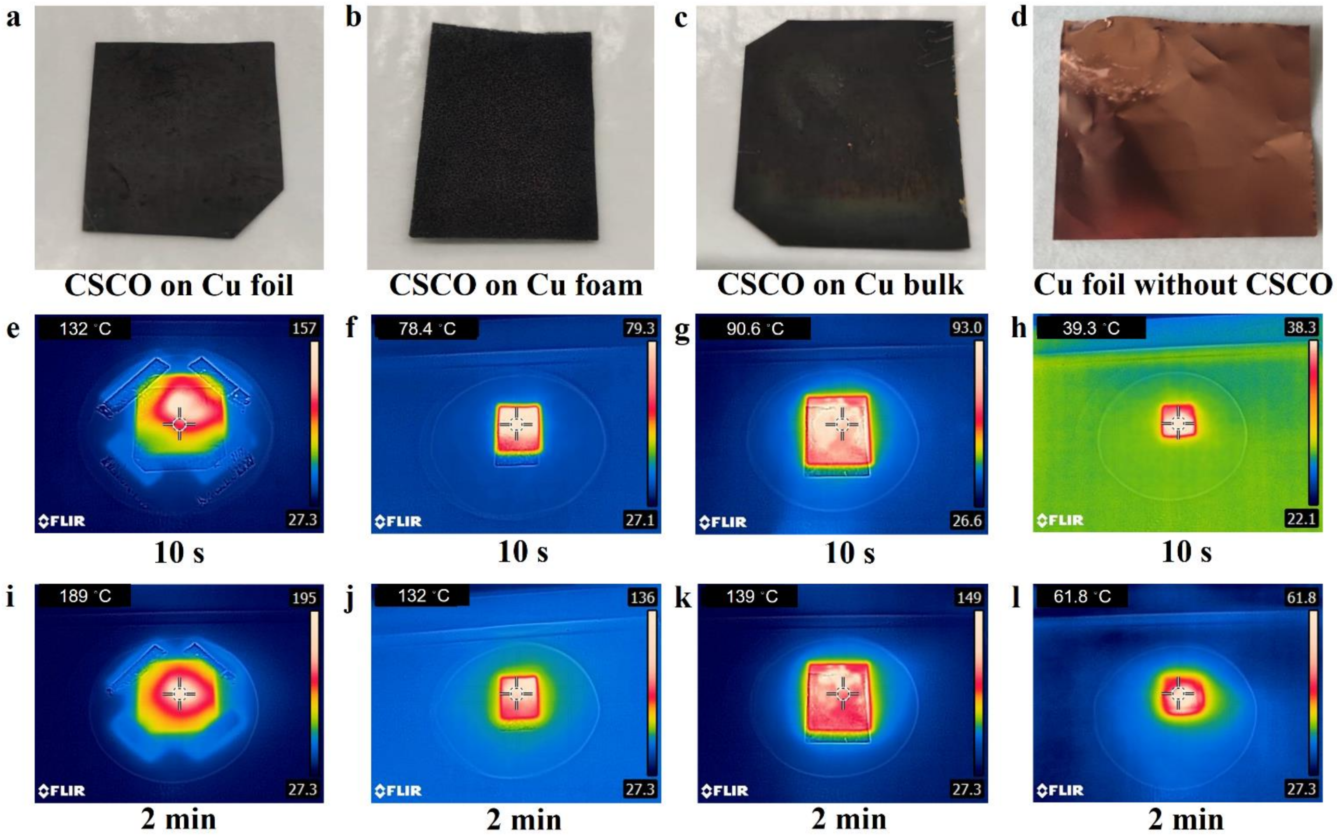

Supplementary Figure 14. Photothermal effect for CSCO HNA *in situ* grown on different Cu substrates. Optical images of CSCO HNA *in situ* grown on (a) Cu foil, (b) Cu foam, (c) Cu bulk, and (d) pure Cu foil without CSCO. Photothermal images after 10 s irradiation for CSCO HNA *in situ* grown on (e) Cu foil, (f) Cu foam, (g) Cu bulk, and (h) pure Cu foil without CSCO. Photothermal images after 2 mins irradiation for CSCO HNA *in situ* grown on (i) Cu foil, (j) Cu foam, (k) Cu bulk, and (l) pure Cu foil

without CSCO.

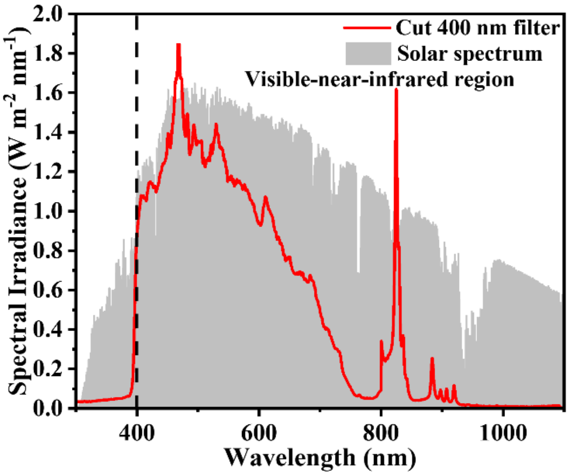

Supplementary Figure 15. The illumination spectrum of our light simulator comparing with sunlight.

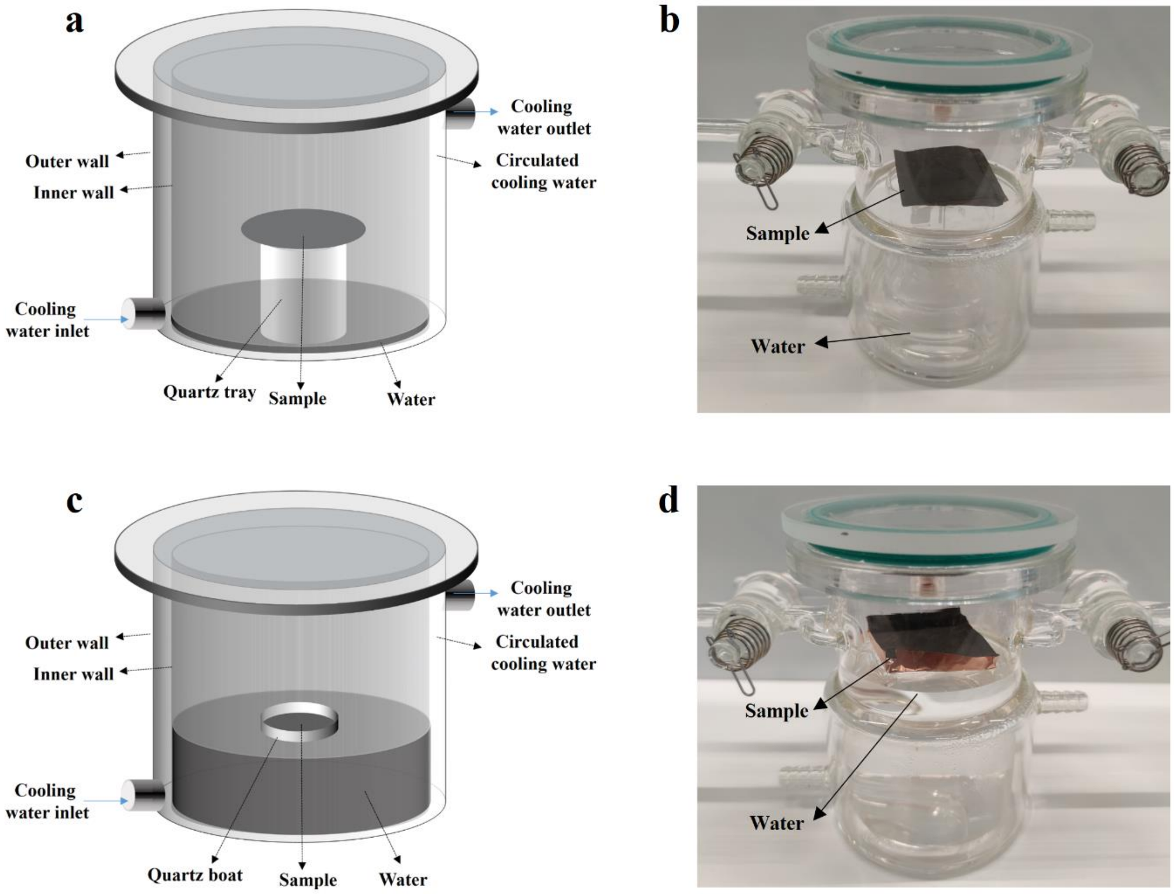

Supplementary Figure 16. The setup of photocatalytic reactor. (a) Schematic diagram and (b) digital image of reactor for photothermal catalysis in our work; (c) schematic diagram and (d) digital image of reactor for pure photocatalysis excluding the heat, in which the Cu-CSCO HNA film floats directly on the water and can quickly conduct localized heat to maintain its room temperature.

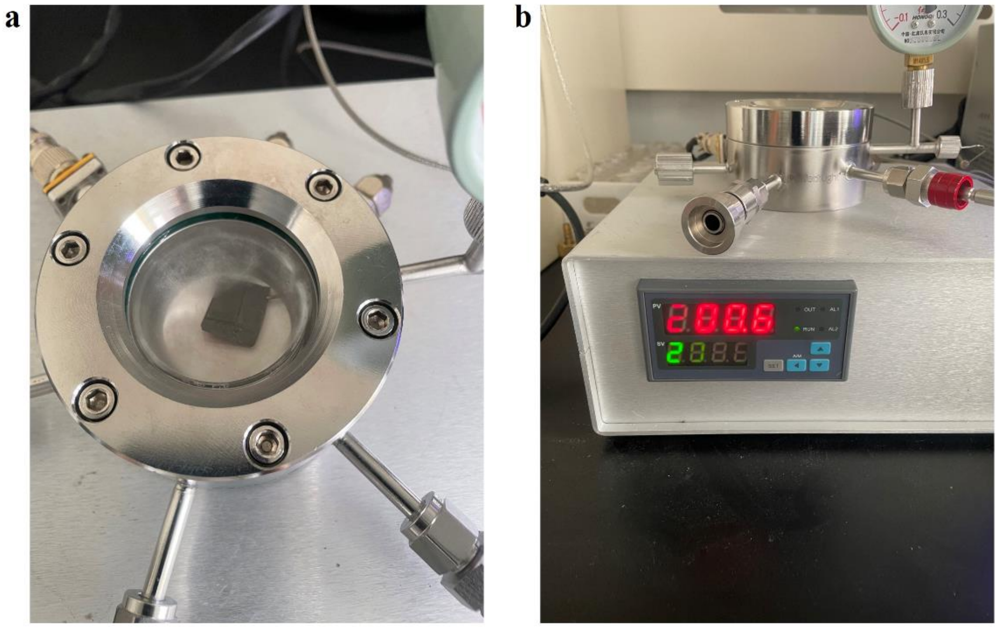

Supplementary Figure 17. Thermocatalysis using Cu-CSCO HNA. (a) Digital image of thermocatalytic reactor; (b) the temperature controller and corresponding reaction temperature during the thermocatalysis. Note: a ~200℃ temperature was applied by a thermocouple, and no light irradiation.

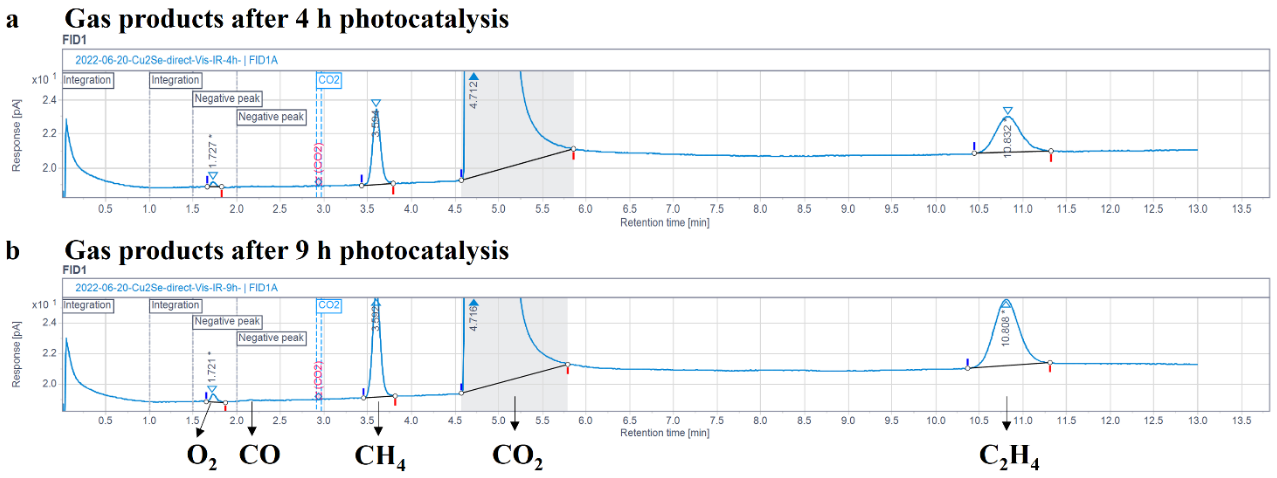

Supplementary Figure 18. Gas products detected by GC after (a) 4h and (b) 9h photocatalysis over Cu-CSCO HNA.

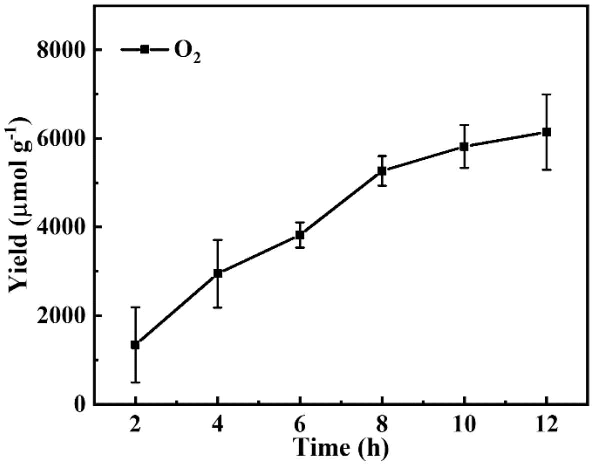

Supplementary Figure 19. The detected yield of O_2 by GC during photothermal catalysis Cu-CSCO HNA with irradiation time. The obtained yield for O_2 generation is around 632.96 μmol g^{-1} h^{-1}. It is worth noting that the consumed holes calculated from oxygen yield are smaller than the electrons consumed by CO_2 reduction. That's because a lot of the oxygen produced will be dissolved in the solution and difficult to detect.

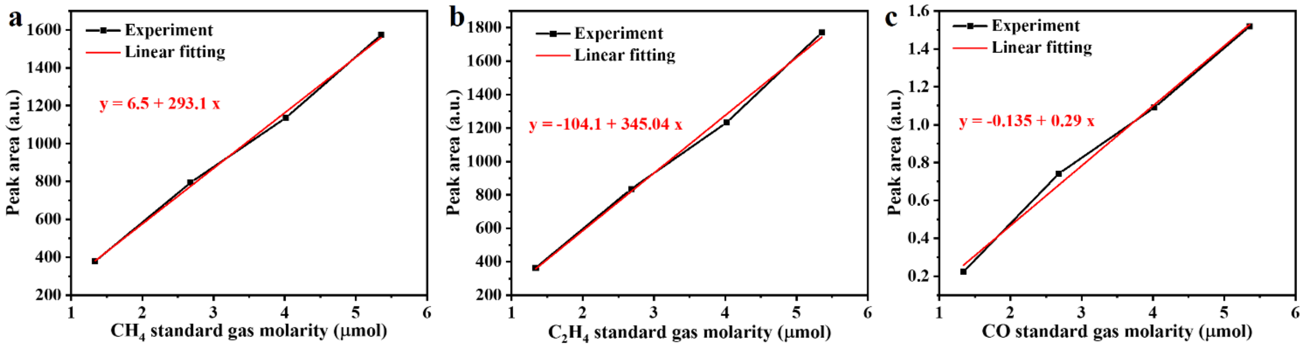

Supplementary Figure 20. The standard curves of (a) CH_4, (b) C_2H_4 and (c) CO for calculation gas products after photocatalysis.

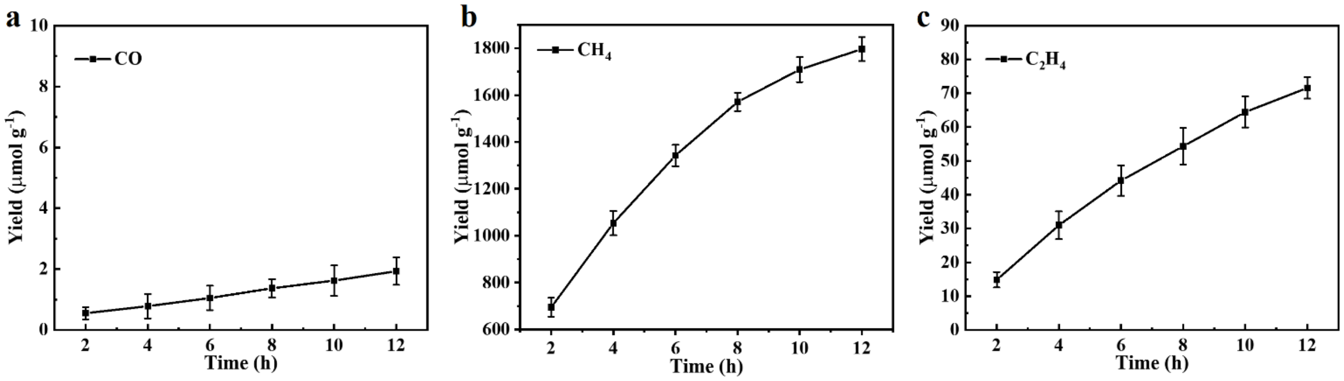

Supplementary Figure 21. The yield of (a) CO, (b) CH_4 and (c) C_2H_4 for photothermal CO_2 conversion over Cu-CSCO HNA with irradiation time. 2 × 3 cm^2 Cu foil with 9.12 mg loading CSCO HNA is used for the photothermal catalysis.

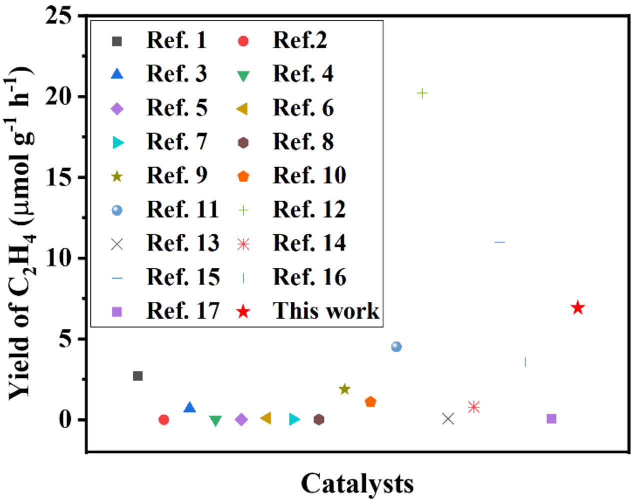

Supplementary Figure 22. Performance comparison of C_2H_4 yield over Cu-CSCO HNA with previous reports. All the performance in the references is obtained under photocatalysis, it clearly shows that the yield of C_2H_4 generated by Cu-CSCO HNA outperforms most of reported catalysts.[1, 2, 3, 4, 5, 6, 7, 8, 9, 10, 11, 12, 13, 14, 15, 16, 17]

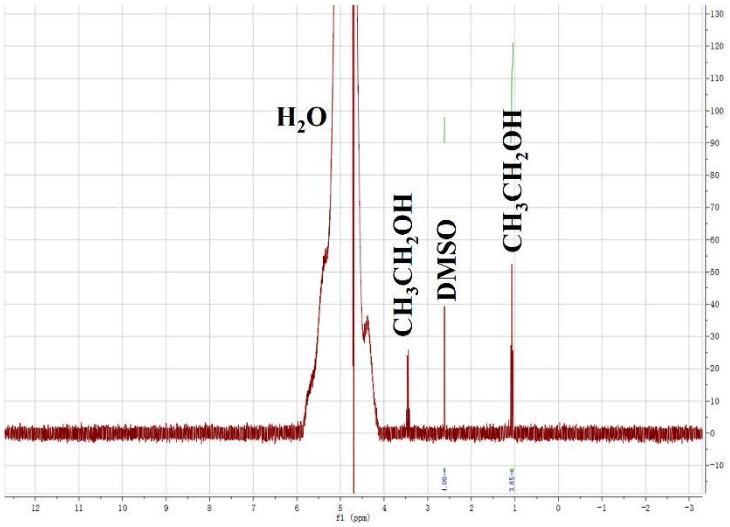

Supplementary Figure 23. Representative ^{1}H NMR spectrum of the liquids after 6 h CO_2 photocatalysis over Cu-CSCO HNA. The liquid products were quantified by nuclear magnetic resonance (NMR) (Bruker AVANCE AV III 400) spectroscopy, in which dimethyl sulfoxide (DMSO, Sigma, 99.99%) was used as the internal standard.

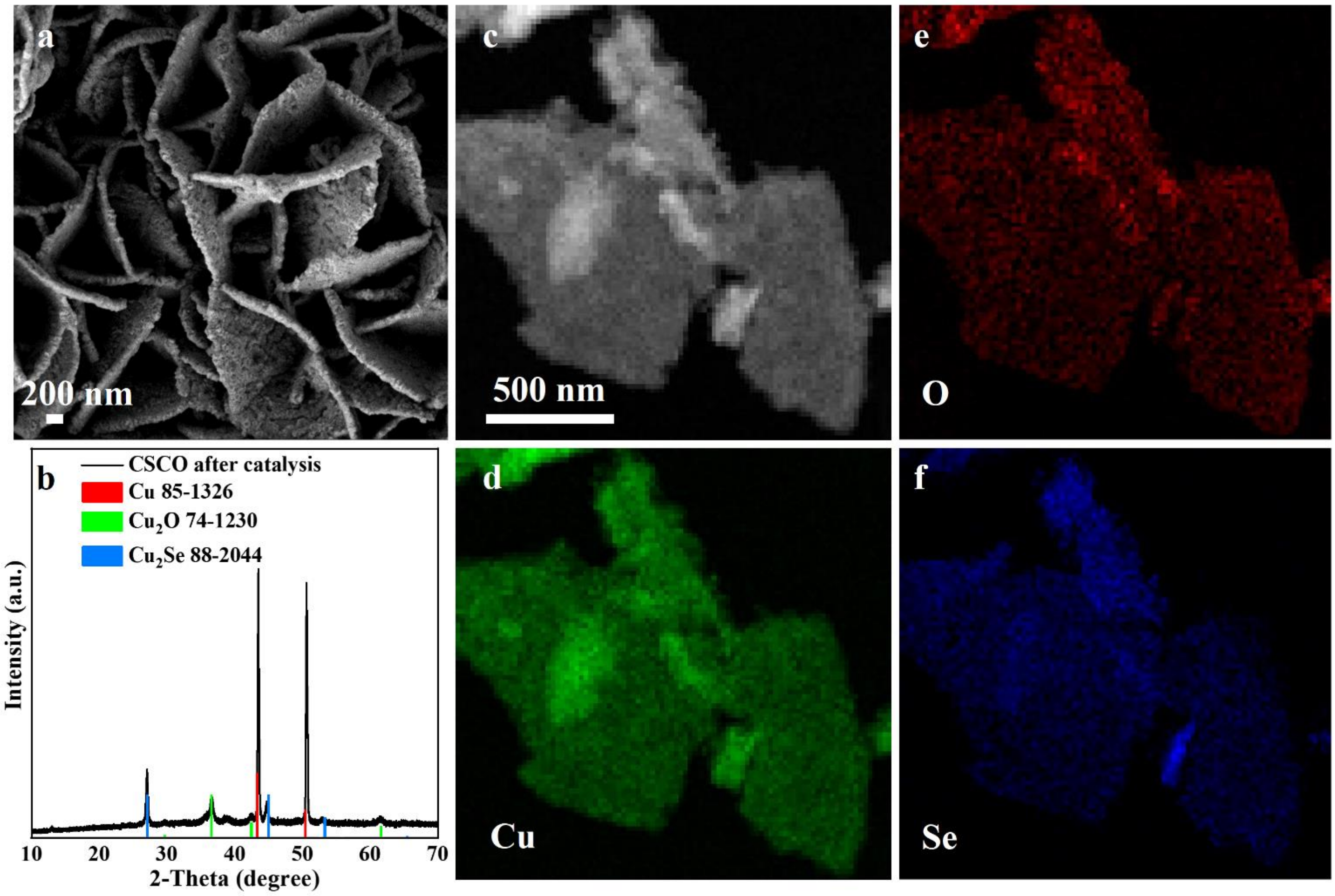

Supplementary Figure 24. Characterizations of Cu-CSCO HNA after 10 cycles of photothermal CO_2 reduction. (a) SEM images; (b) XRD patterns; (c)-(f) STEM and EDS mapping images.

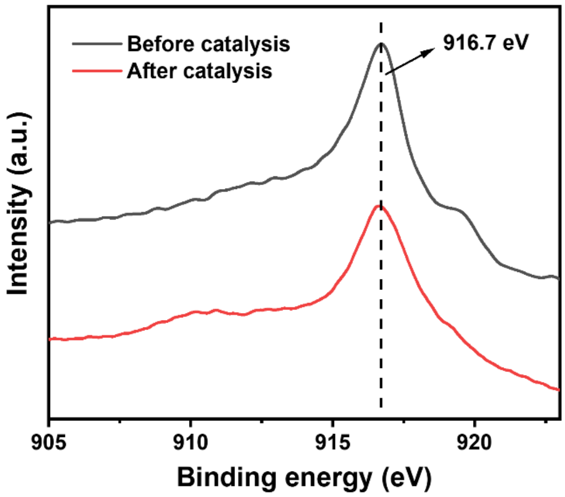

Supplementary Figure 25. Cu LMM Auger electron spectroscopy of Cu-CSCO HNA before (black) and after (red) photothermal catalysis, in which only the peaks of Cu^+ were detected at around 916.7 eV.

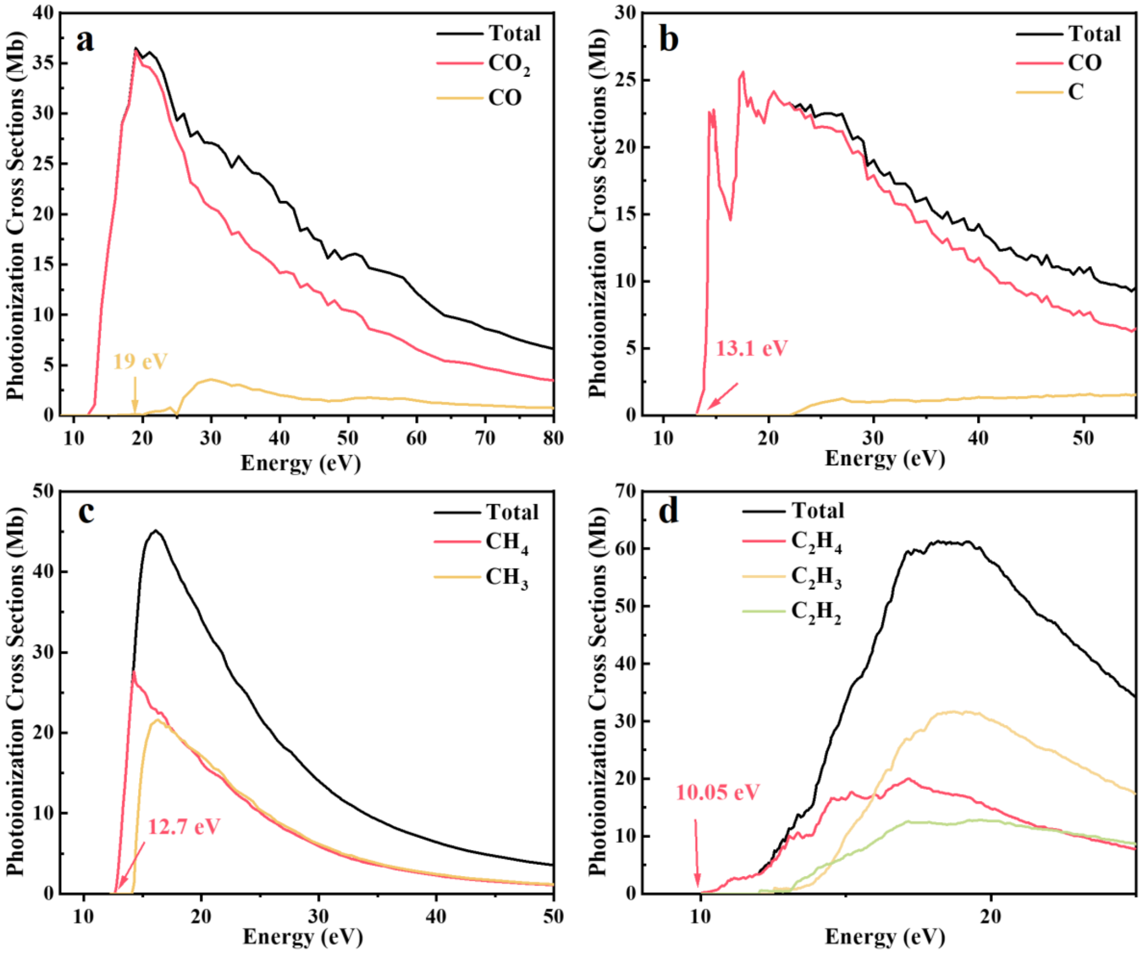

Supplementary Figure 26. Synchrotron-based vacuum ultraviolet photoionization mass spectrometry (SVUV-PIMS). Absolute photoionization cross sections for (a) CO_2, (b) CO, (c) CH_4 and (d) C_2H_4. *http://flame.nsrl.ustc.edu.cn/database/data.php* **Supplementary Figure 26a** reveals that CO_2 would dissociate into CO when the photon energy approaches about 19 eV; meanwhile, the pure CO and CH_4 can be detected when the photon energy is up to 13.1 eV and 12.7 eV, respectively (**Supplementary Figure 26b-c**). As such, it is feasible to utilize SVUV-PIMS spectra at the photon energy of 14.5 eV for distinguishing whether CO, CH_4 and C_2H_4 is obtained from CO_2 reduction or dissociation.

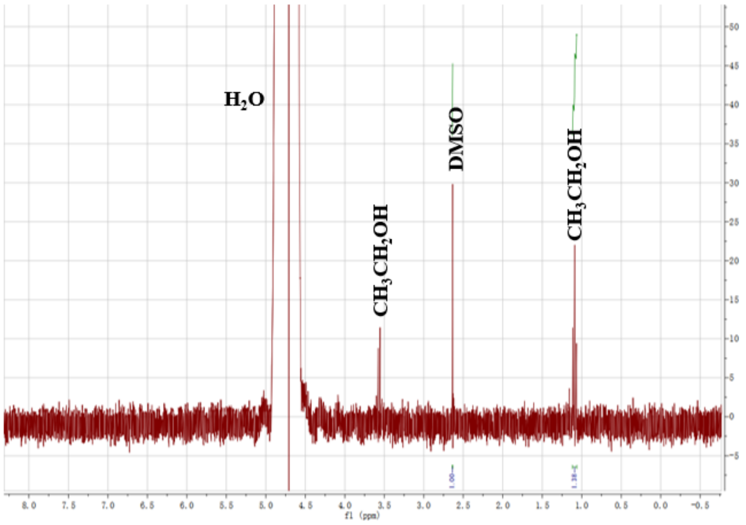

Supplementary Figure 27. Representative ^{1}H NMR spectrum of the liquids over Cu-CSCO HNA after 6 h CO_2 photocatalysis under natural solar spectrum. The liquid products were quantified by nuclear magnetic resonance (NMR) (Bruker AVANCE AV III 400) spectroscopy, in which dimethyl sulfoxide (DMSO, Sigma, 99.99%) was used as the internal standard.

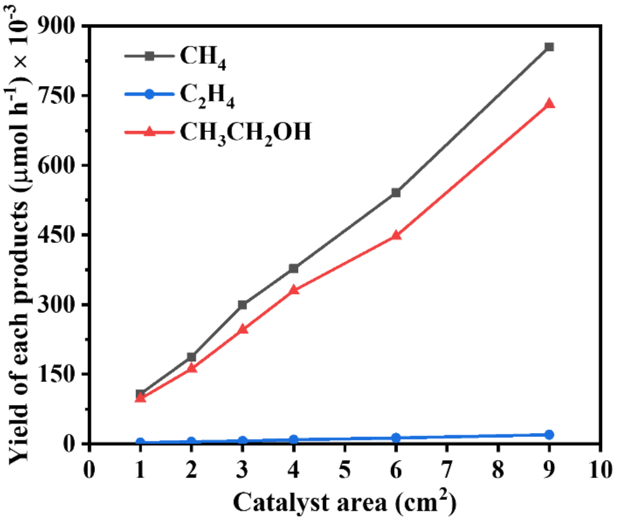

Supplementary Figure 28. Product yield of Cu-CSCO HNA for CO_2 photoconversion under solar spectrum with different catalyst area, including 1 × 1 cm^2, 1 × 2 cm^2, 1 × 3 cm^2, 2 × 2 cm^2, 2 × 3 cm^2, 3 × 3 cm^2.

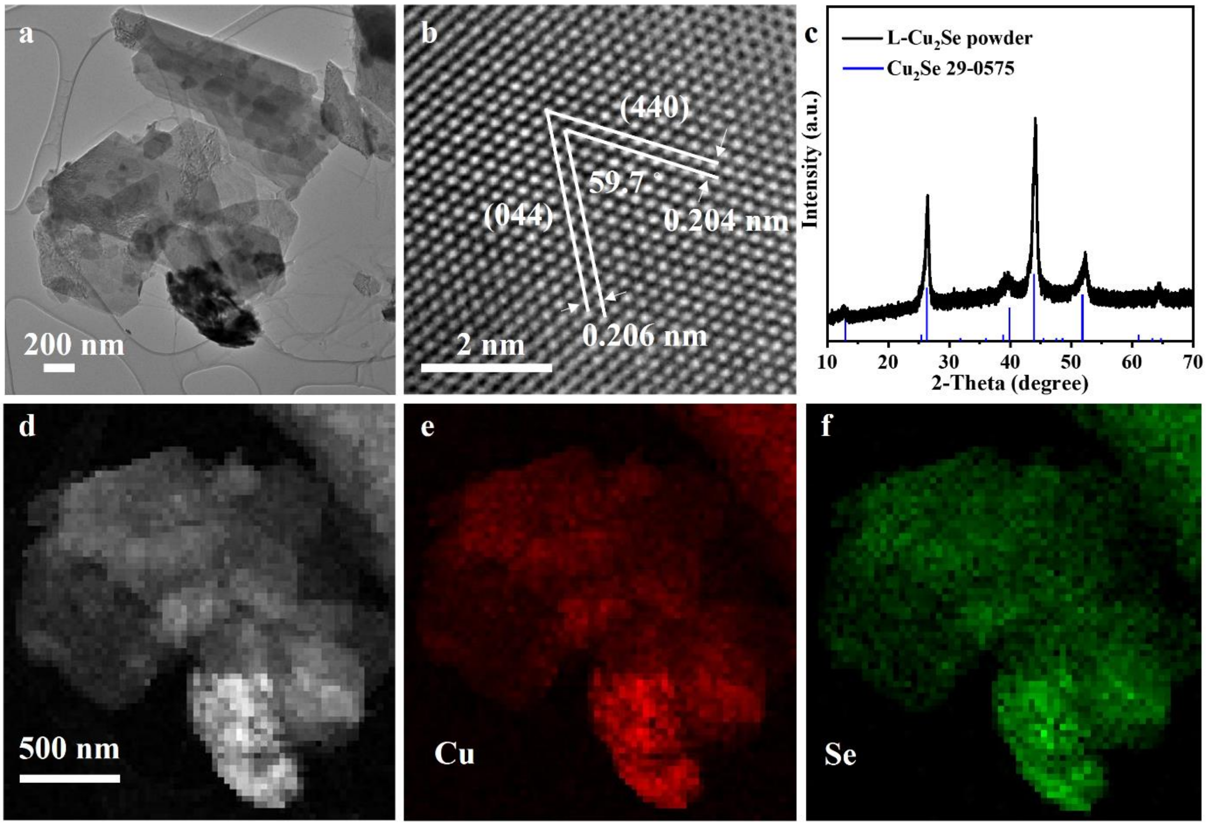

Supplementary Figure 29. Characterizations of L-Cu_2Se nanosheet powder. (a) TEM image; (b) HRTEM image, in which the exposed facet can be inferred along [111] direction because 0.204 nm and 0.206 nm interplanar distances match well with the d_{440} and d_{044} spacings, and the corresponding dihedral angle of 59.7° agrees well with the calculated angle between the (440) and (044) planes; (c) XRD patterns; (d)-(f) annular dark-field TEM images and corresponding elemental mapping images. The initially obtained Cu_2Se nanosheets are also identified as L-Cu_2Se. TEM and HRTEM images confirm the obtained Cu_2Se nanosheets have a flake-like morphology with hexagonal crystallinity. ESD mapping images show their even distribution of Cu and Se elements.

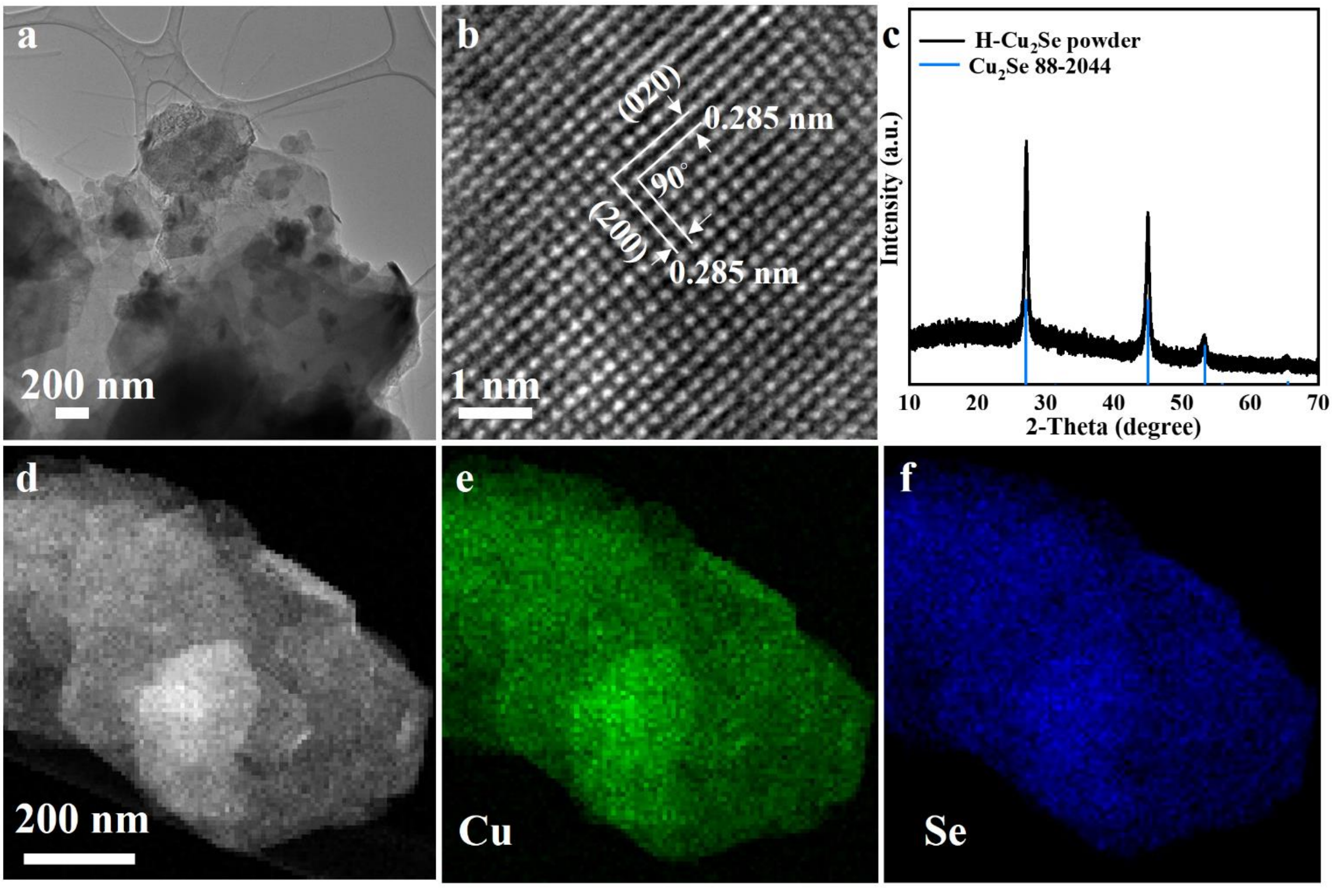

Supplementary Figure 30. Characterizations of H-Cu_2Se nanosheet powder. (a) TEM image; (b) HRTEM image, in which the exposed facet can be inferred along [001] direction because 0.285 nm and 0.285 nm interplanar distances match well with the d_{020} and d_{200} spacings, and the corresponding dihedral angle of 90° agrees well with the calculated angle between the (020) and (200) planes; (c) XRD patterns; (d)-(f) annular dark-field TEM images and corresponding elemental mapping images. Similar to the Cu_2Se nanosheets *in situ* grown on the Cu foil, the independent L-Cu_2Se nanosheets can be easily converted into the H-Cu_2Se nanosheets by calcination in Ar atmosphere.

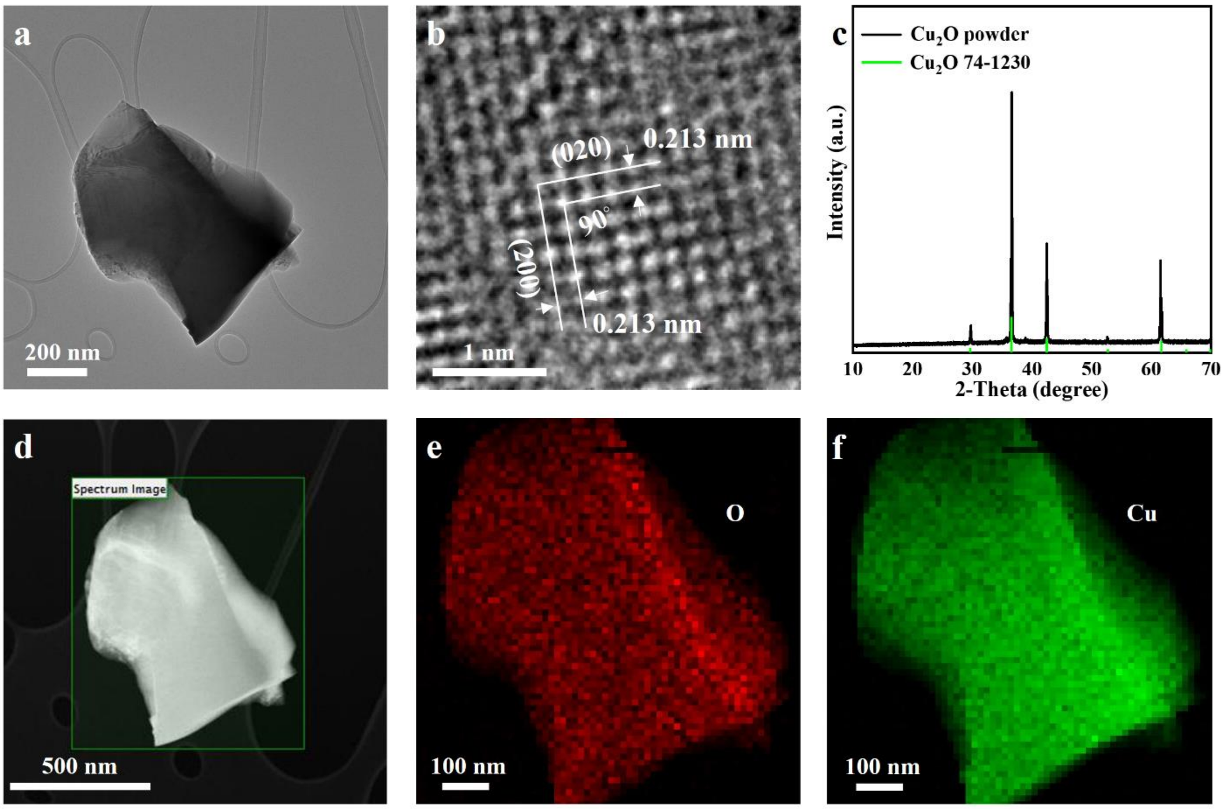

Supplementary Figure 31. Characterizations of commercial Cu_2O powder. (a) TEM image; (b) HRTEM image, in which the exposed facet can be inferred along [001] direction because 0.213 nm interplanar distances match well with the d_{020} and d_{200} spacings, and the corresponding dihedral angle of 90° agrees well with the calculated angle between the (020) and (200) planes; (c) XRD patterns; (d)-(f) annular dark-field TEM images and corresponding elemental mapping images.

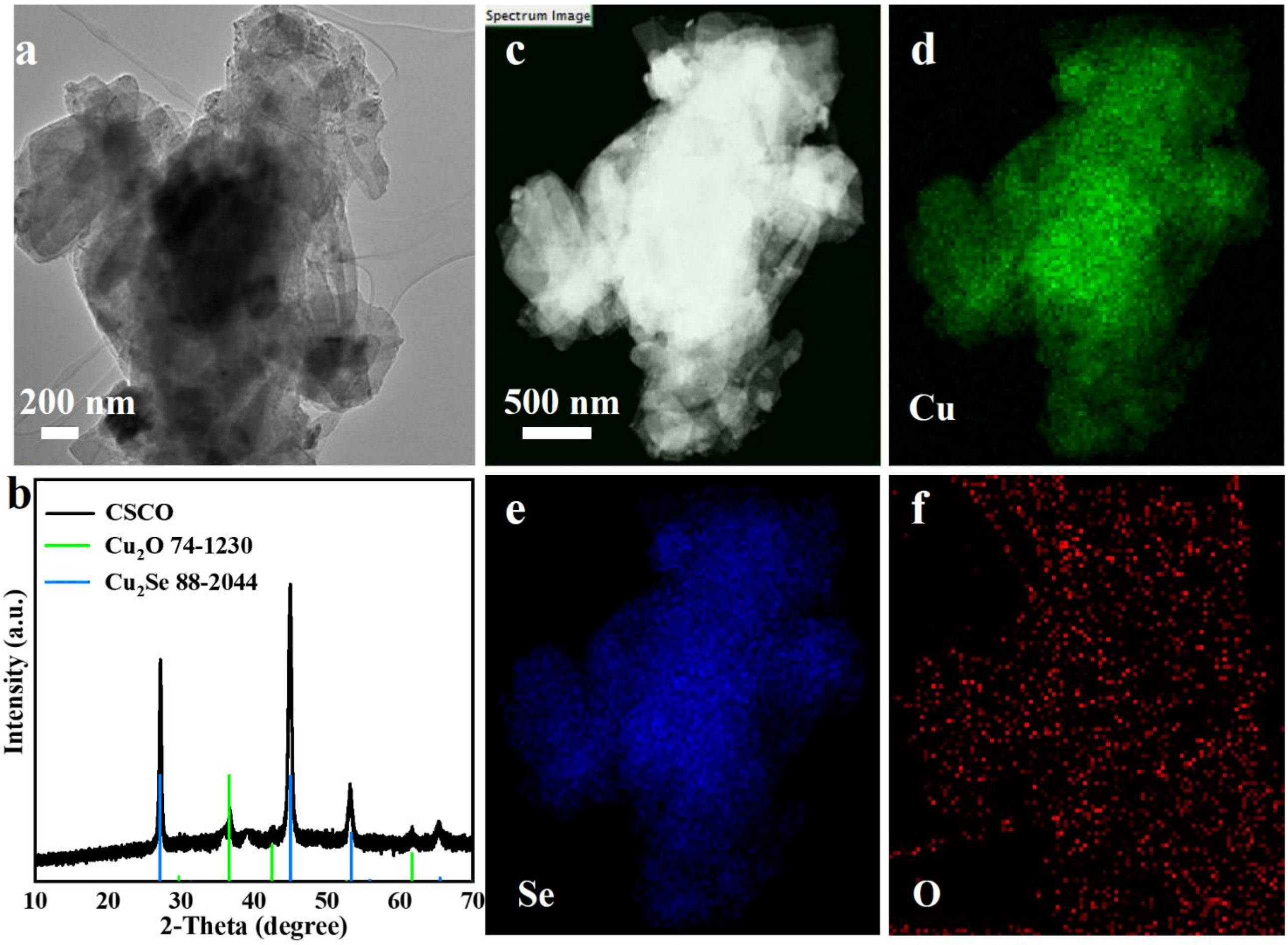

Supplementary Figure 32. Characterizations of independent CSCO heterostructure-nanosheet powder. (a) TEM image; (b) XRD patterns; (c)-(f) annular dark-field TEM images and corresponding elemental mapping images. After continued calcination of the independent Cu_2Se nanosheets in air, CSCO heterostructure powder can be synthesized. TEM images confirm their unchanged 2D configuration. XRD patterns show that there are mixed crystal phases of Cu_2Se (PDF: 88-2044) and Cu_2O (PDF: 74-1230), indicating the coexistence of these two compounds. STEM and element mapping images validate the appearance of oxygen element and the even distribution of Cu and Se, further suggesting the formation of surface Cu_2O particles.

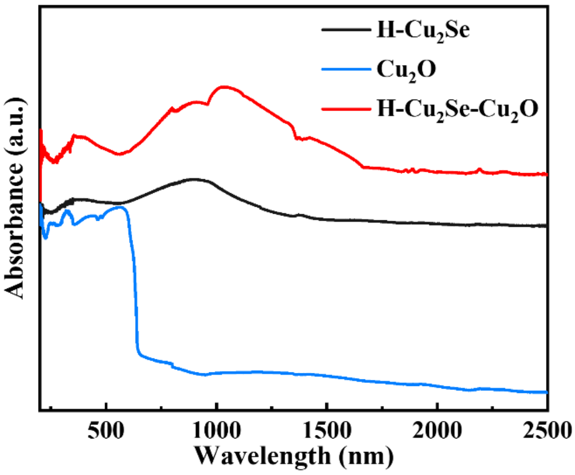

Supplementary Figure 33. UV-vis-NIR diffuse reflectance spectra of independent Cu_2O, H-Cu_2Se and CSCO powders.

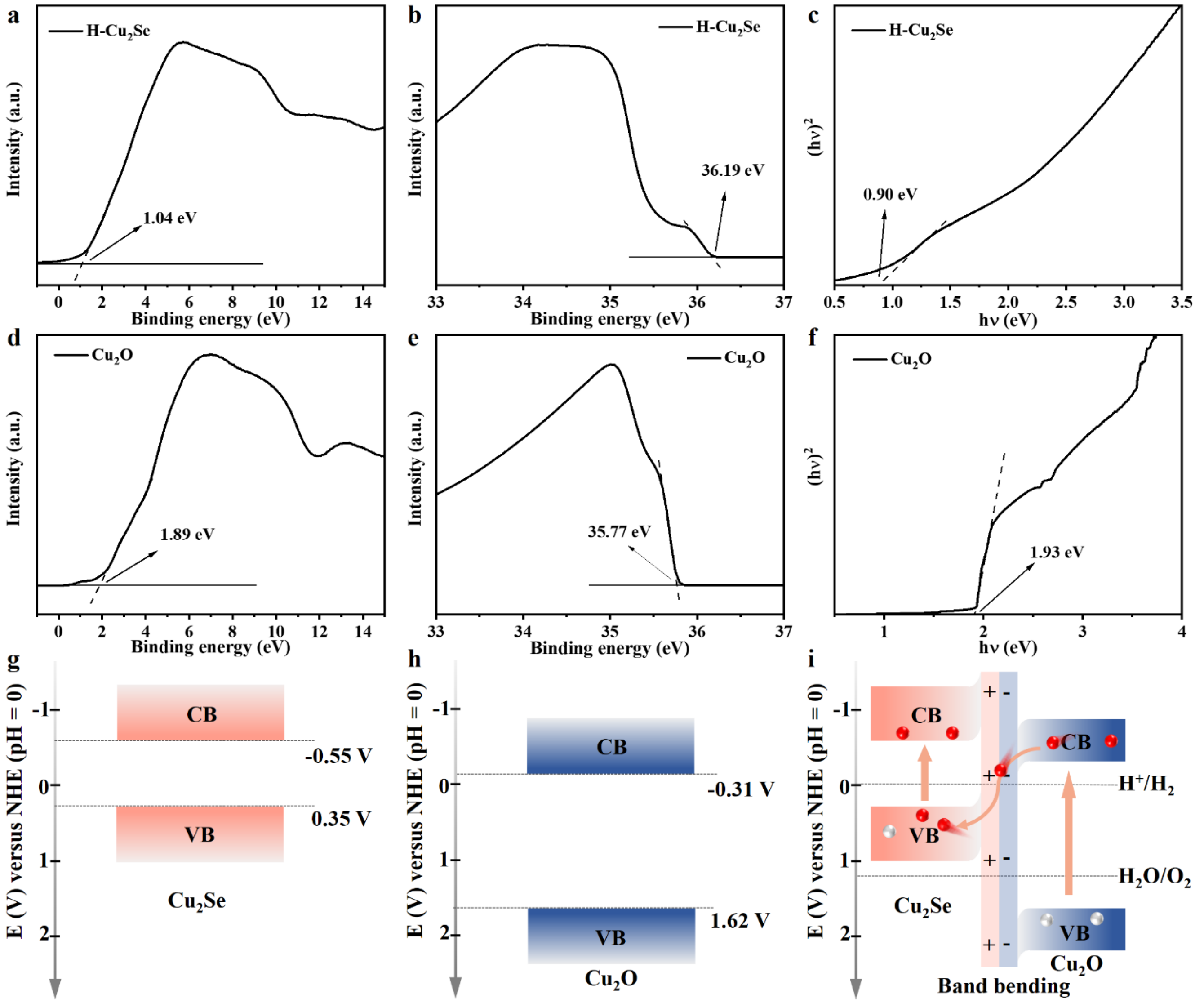

Supplementary Figure 34. UPS and experimental band structure of independent H-Cu_2Se and Cu_2O powder. SRPES valence-band, secondary electron cutoff spectra and Tauc plots for (a)-(c) H-Cu_2Se and (d)-(f) Cu_2O. The corresponding band structure of (g) H-Cu_2Se, (h) Cu_2O and (i) CSCO heterostructure.

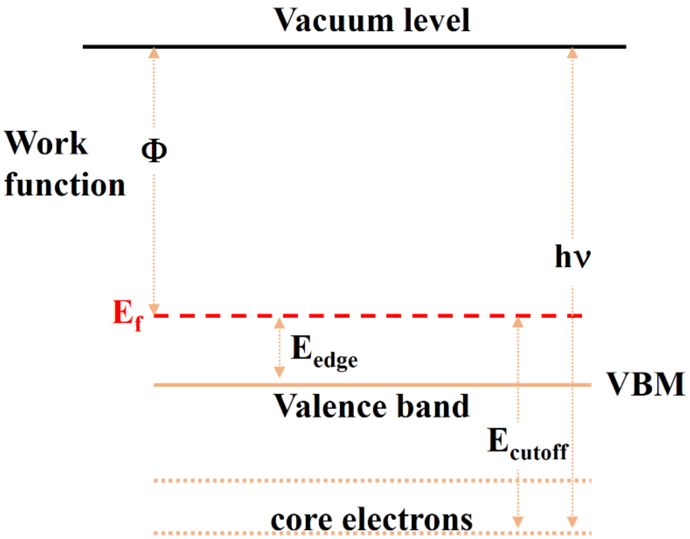

Supplementary Figure 35. Schematic illustration of the energy level information from UPS. The valence band maxima (VBM) of the samples referenced to Normal Hydrogen Electrode (NHE) can be obtained according to the following equations[18, 19]:

$$\Phi = h\nu - E_{cutoff}$$

$$E_{VBM} = E_{edge} + \Phi - 4.5\ (vs.\ \text{NHE, pH} = 0)$$

where Φ is the work function, hν is the photon energy of the excitation source (hν = 40 eV in our work), E_{cutoff} is the energy of secondary electron cutoff, and E_{VBM} is the valence band maxima of samples *vs.* NHE at pH = 0.

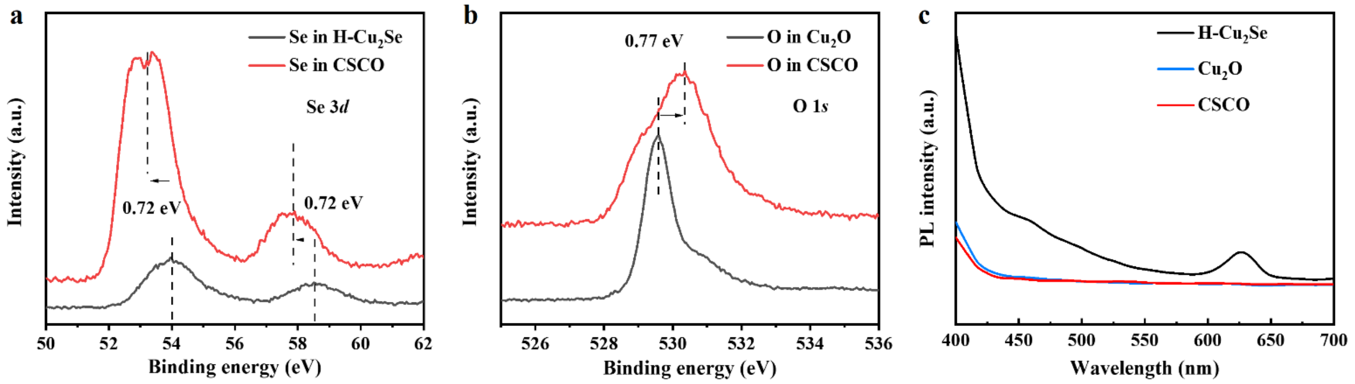

Supplementary Figure 36. XPS spectra and photoluminescence (PL) spectra of independent H-Cu_2Se, Cu_2O and CSCO. (a) High-resolution Se 3*d* spectra of H-Cu_2Se and CSCO powder; (b) high-resolution O 1*s* spectra of Cu_2O and CSCO powder. One can clearly see that Se 3*d* peaks shift towards the low energy while O 1*s* peak shifts towards the high energy after forming the CSCO heterostructure, which demonstrates that the electrons are transferred from Cu_2O to Cu_2Se in the CSCO heterostructure. (c) Room-temperature PL spectra, in which the CSCO powder exhibits the lowest PL signal, indicating the best performance for charge carrier separation compared with the pure H-Cu_2Se and Cu_2O.

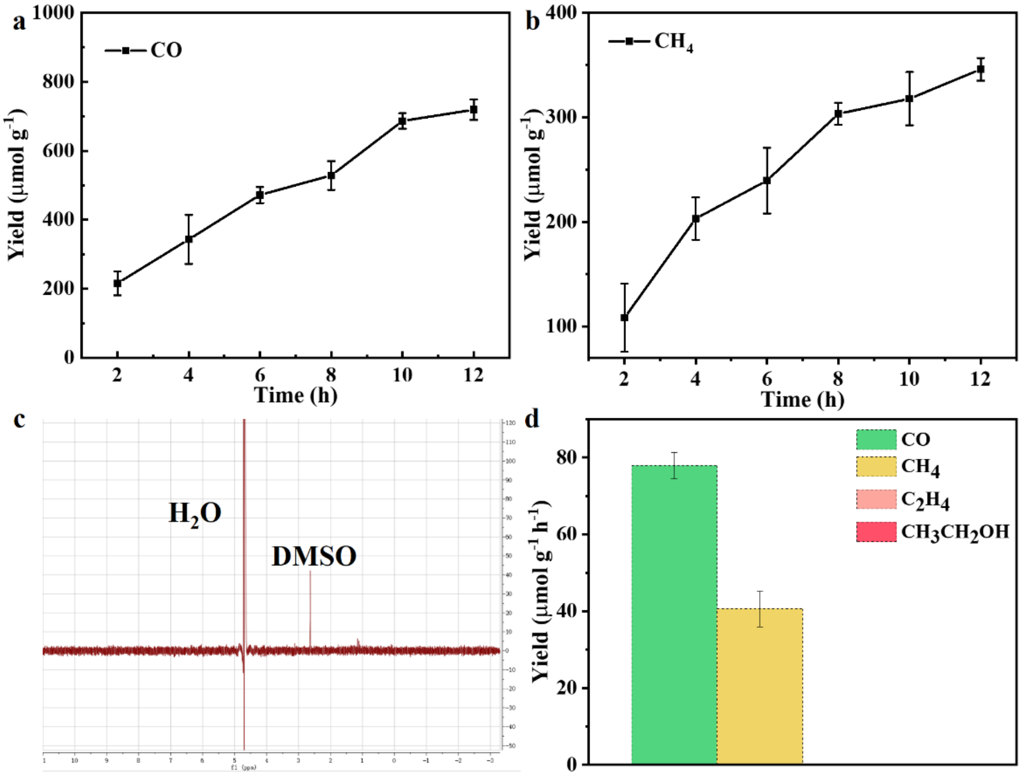

Supplementary Figure 37. The performance of CSCO heterostructure-nanosheet powder sprayed on the quartz (CSCO-Q). The yield of (a) CO and (b) CH_4 with reaction time; (c) ^1H NMR spectrum of liquid products after 12 h reaction; (d) the generation rate of various products over this catalytic system. In this control experiment, mass loading of CSCO heterostructure-nanosheet powder is calculated to 1.52 mg cm^{-2}, the same with that obtained in the Cu-CSCO HNA.

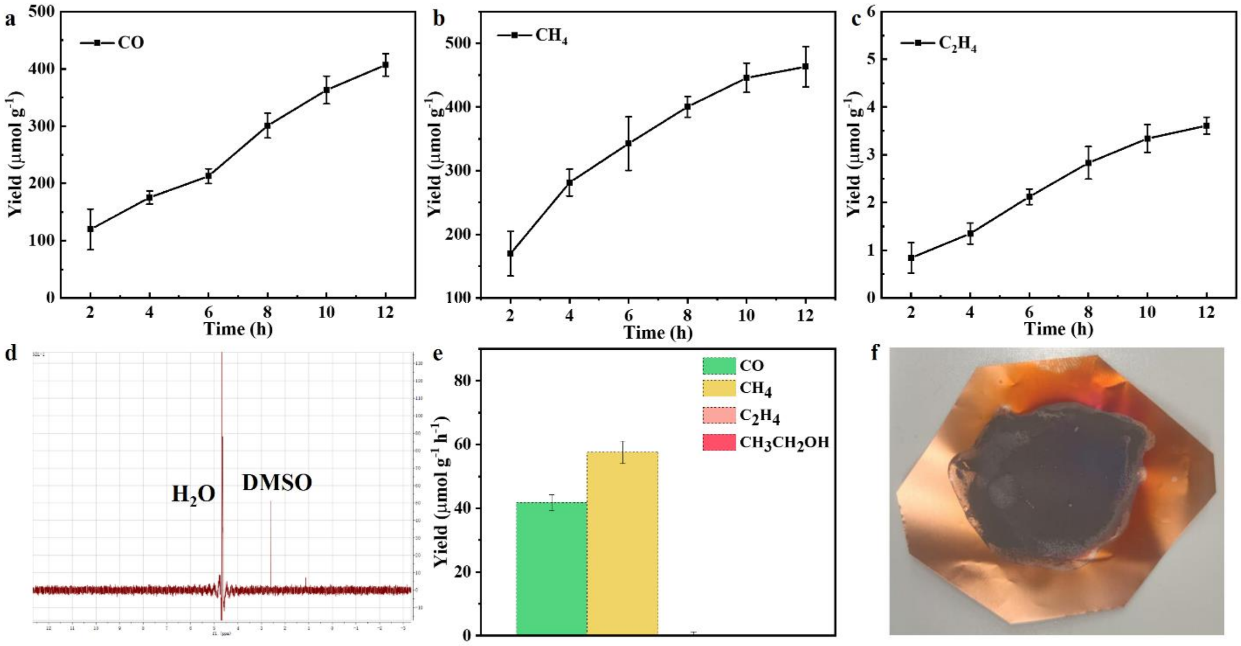

Supplementary Figure 38. The performance of CSCO heterostructure-nanosheet powder sprayed on the Cu foil (CSCO-C). The yield of (a) CO, (b) CH_4 and (c) C_2H_4 with reaction time; (d) ^1H NMR spectrum of liquid products after 12 h reaction; (e) the generation rate of various products over this catalytic system; (f) digital images of CSCO heterostructure-nanosheet powder spraying on the Cu foil. In this control experiment, mass loading of CSCO heterostructure-nanosheet powder is calculated to 1.52 mg cm^{-2}, the same with that obtained in the Cu-CSCO HNA.

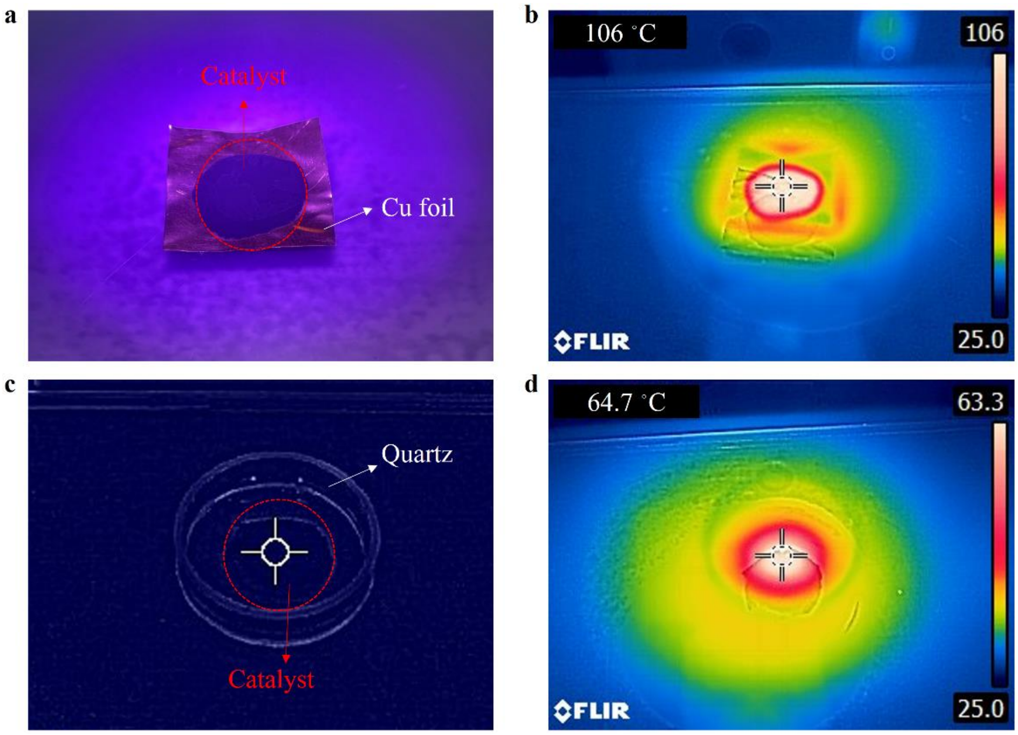

Supplementary Figure 39. Photothermal effect of CSCO powder sprayed on Cu foil (CSCO-C) and quartz (CSCO-Q). (a)-(b) optical and photothermal images after 2 mins irradiation for CSCO-C; (c)-(d) optical and photothermal images after 2 mins irradiation for CSCO-Q. CSCO-C exhibits much higher

temperature than that in the CSCO-Q under the solar irradiation, indicating that the Cu foil can efficient avoid the heat dissipation and retain the localized high temperature.

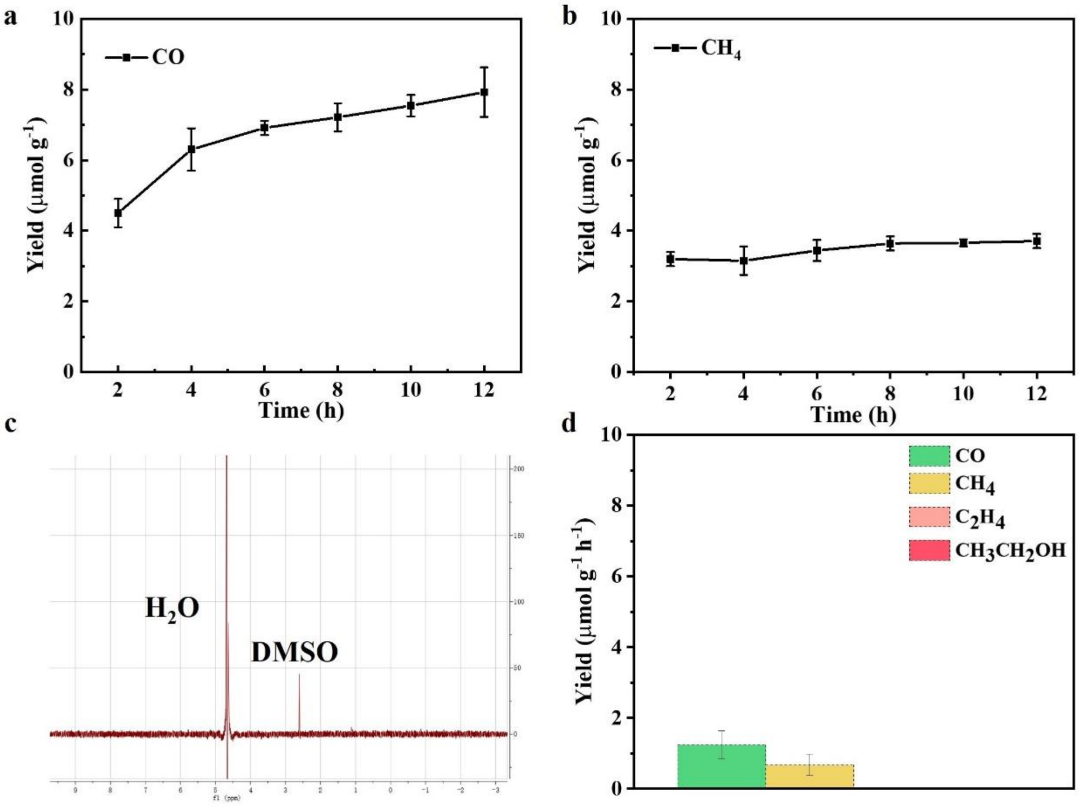

Supplementary Figure 40. The performance of mechanically mixed catalytic system of Cu_2Se nanosheet and commercial Cu_2O powder. The yield of (a) CO and (b) CH_4 with reaction time; (c) ^1H NMR spectra of liquid products after 12 h reaction; (d) the generation rate of various products over this catalytic system.

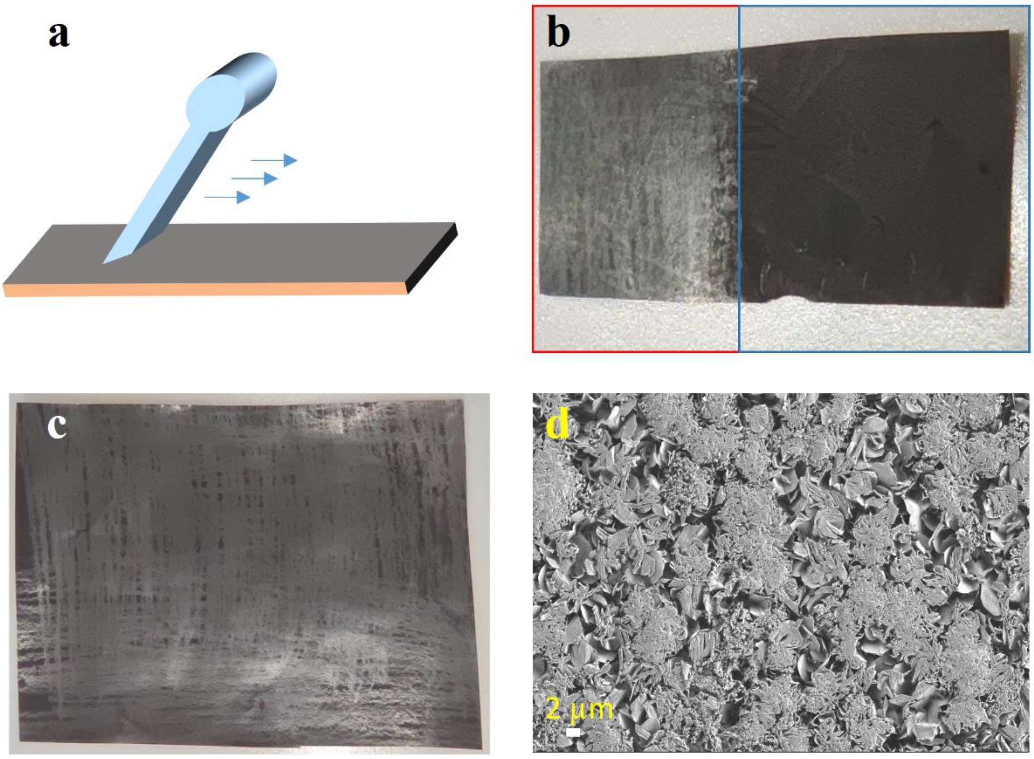

Supplementary Figure 41. Cu-CSCO HNA treated by mechanical friction. (a) The scheme of the mechanical friction process. (b) Digital images of Cu-CSCO HNA with (blue box) and without gap (red box). (c) Digital images of Cu-CSCO HNA without gap for catalysis experiment. (d) SEM image.

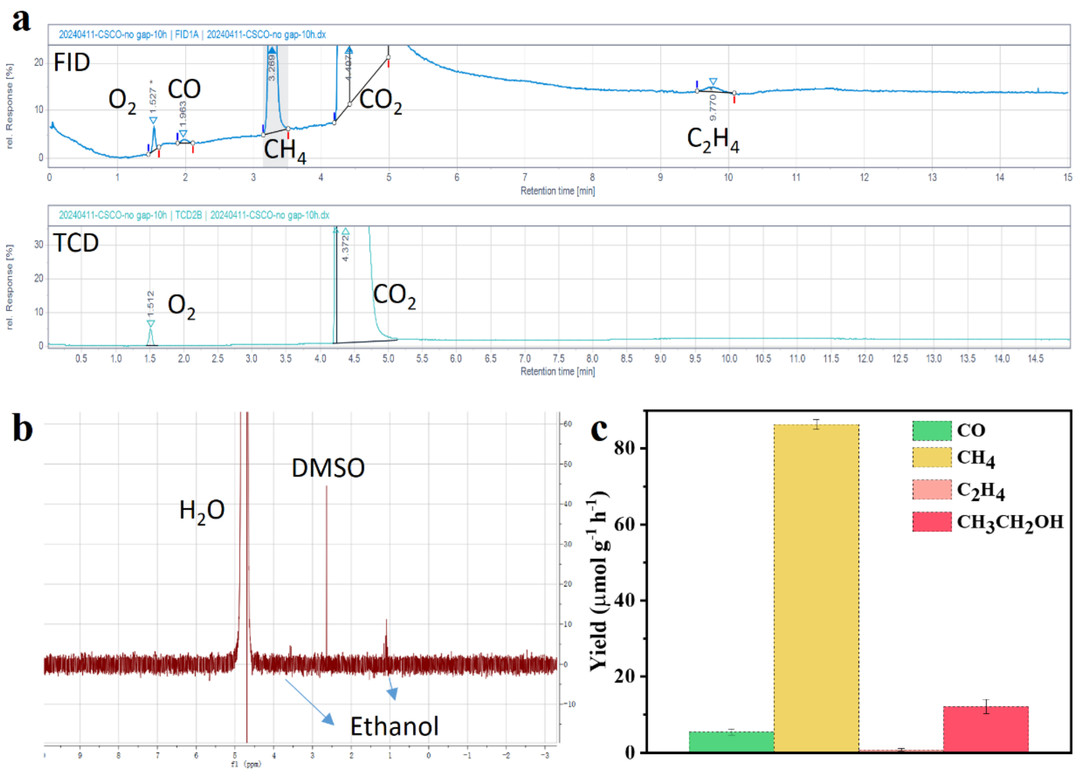

Supplementary Figure 42. The performance of Cu-CSCO HNA without gap for photothermal CO_2 reduction. (a) GC of the gas products, in which the CO, CH_4 and C_2H_4 were detected as the reduction

products while the O_2 was the oxidation product. (b) ^1H NMR spectrum for liquid product, in which DMSO was used as the reference. (c) The yield of each product (CO: 5.44 μmol g^{-1} h^{-1}; CH_4: 86.32 μmol g^{-1} h^{-1}; C_2H_4: 0.81 μmol g^{-1} h^{-1}; ethanol: 12.17 μmol g^{-1} h^{-1}).

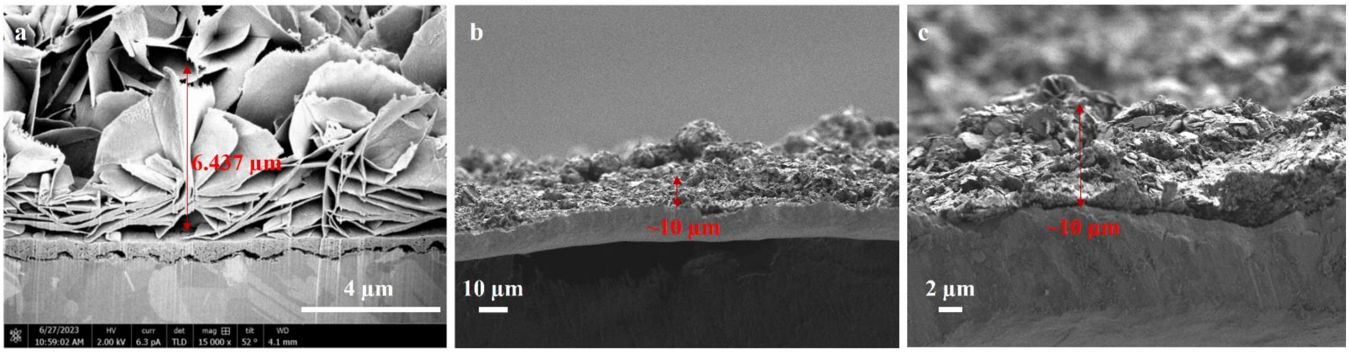

Supplementary Figure 43. SEM cross section images. (a) CSCO HNA *in situ* grown on Cu foil (Cu-CSCO); (b)-(c) CSCO heterostructure-nanosheet powder sprayed on Cu foil (CSCO-C). A distinction between the sprayed and *in situ* grown systems lies in the presence of numerous spatial gaps between the vertically arranged Cu_2Se nanosheets and an increased abundance of Cu-Cu_2Se interfaces within the *in situ* grown Cu-CSCO system.

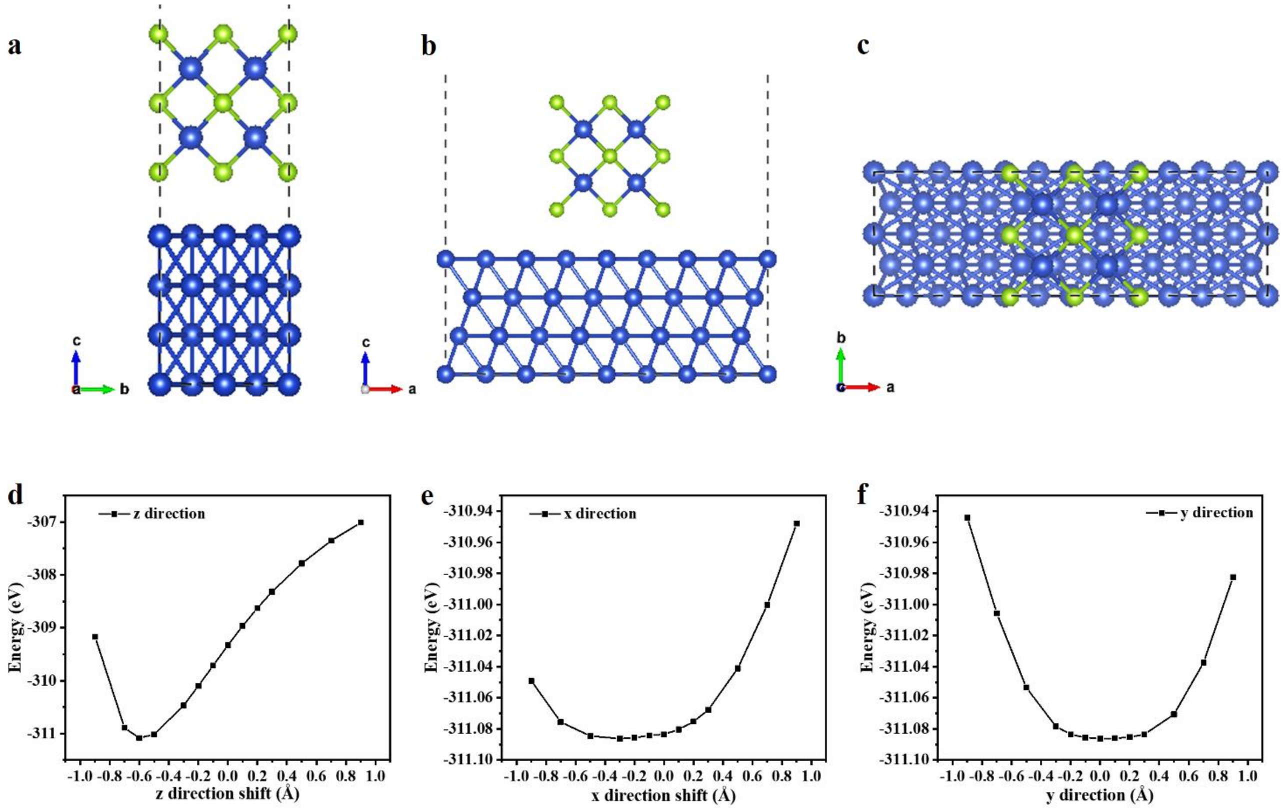

Supplementary Figure 44. The initial structure models and energy changes during the optimization processes. (a)-(c) Views of initial models of Cu_2Se nanosheet on Cu foil along different directions; (d)-(f) total energy changes of the structure during optimization along x, y and z directions.

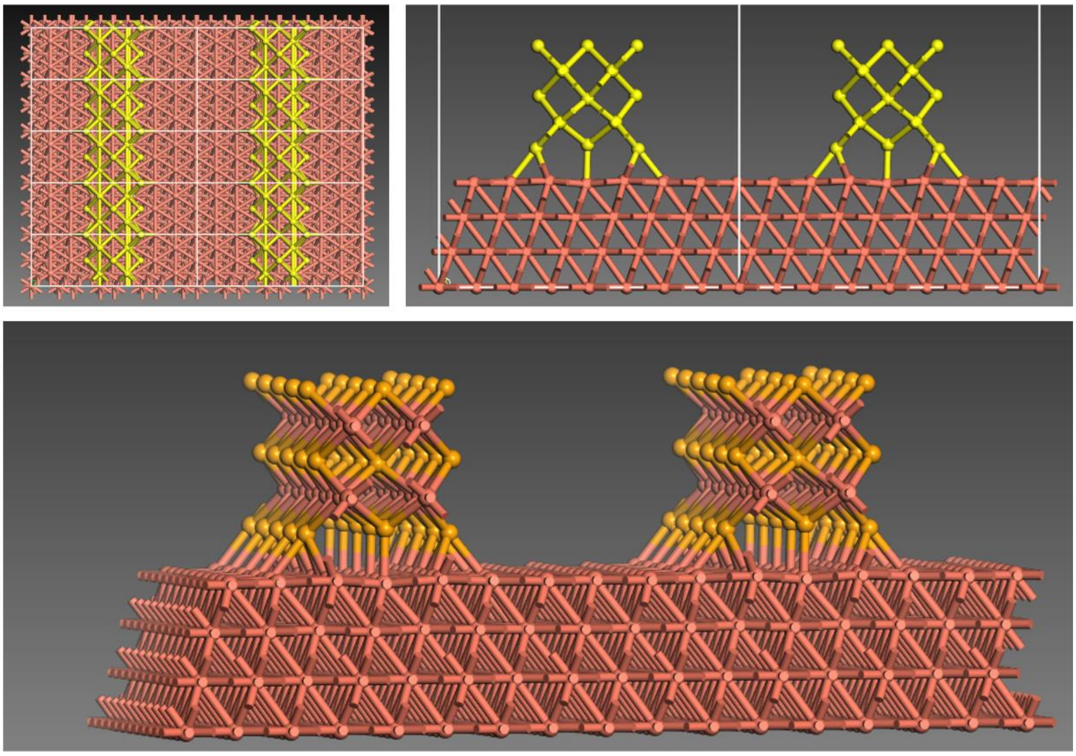

Supplementary Figure 45. The optimized theoretical models of Cu_2Se nanosheet on Cu foil.

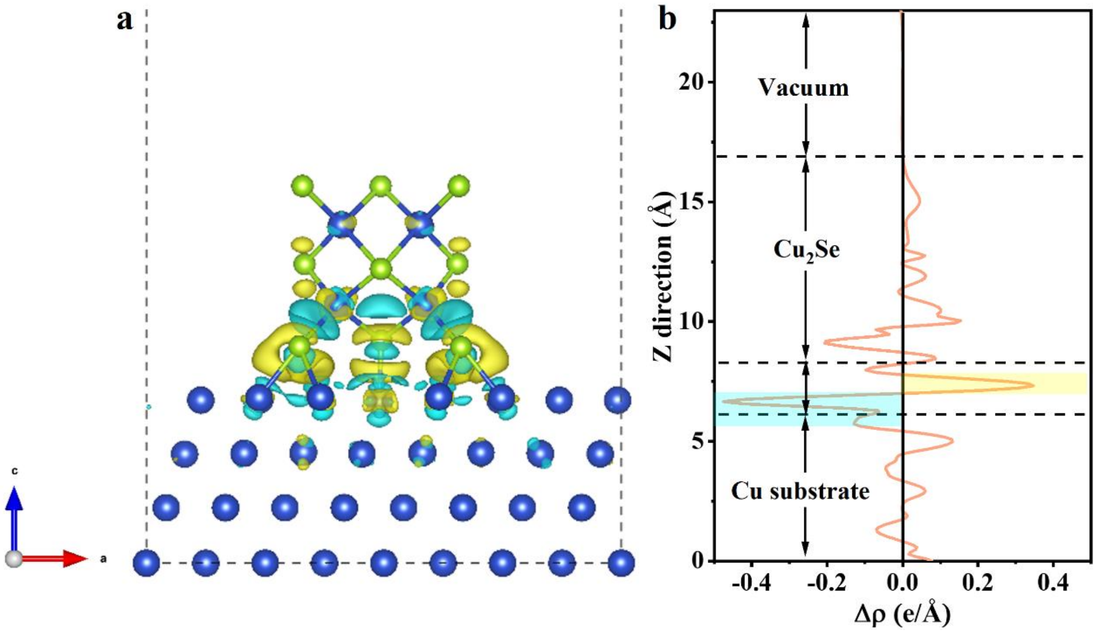

Supplementary Figure 46. The charge density difference (CDD) of Cu-Cu_2Se nanosheet array model. (a) Three dimensional distribution; (b) one dimensional distribution. The yellow and blue isosurfaces correspond to the increase in the number of electrons and the depletion zone, respectively. The isosurfaces are 0.002 e $Bohr^{-3}$.

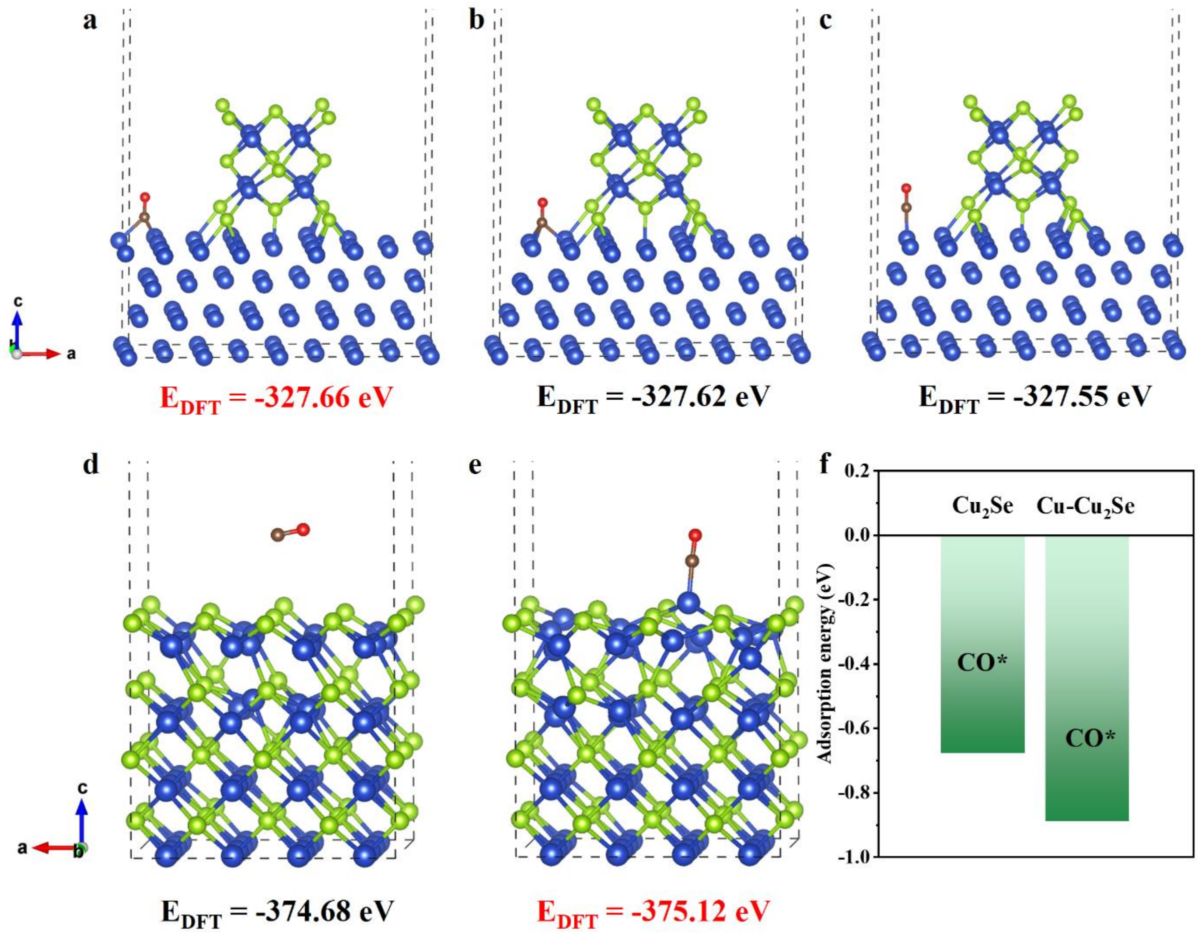

Supplementary Figure 47. CO adsorption energy on different slab models. (a)-(c) Theoretical models and DFT calculated energy (E_{DFT}) of CO adsorbed on the interface of Cu-Cu_2Se; (d)-(e) theoretical models and DFT calculated energy (E_{DFT}) of CO adsorbed on the surface of Cu_2Se; (f) the optimized CO adsorption energy on Cu_2Se (-0.68 eV) and Cu-Cu_2Se (-0.89 eV).

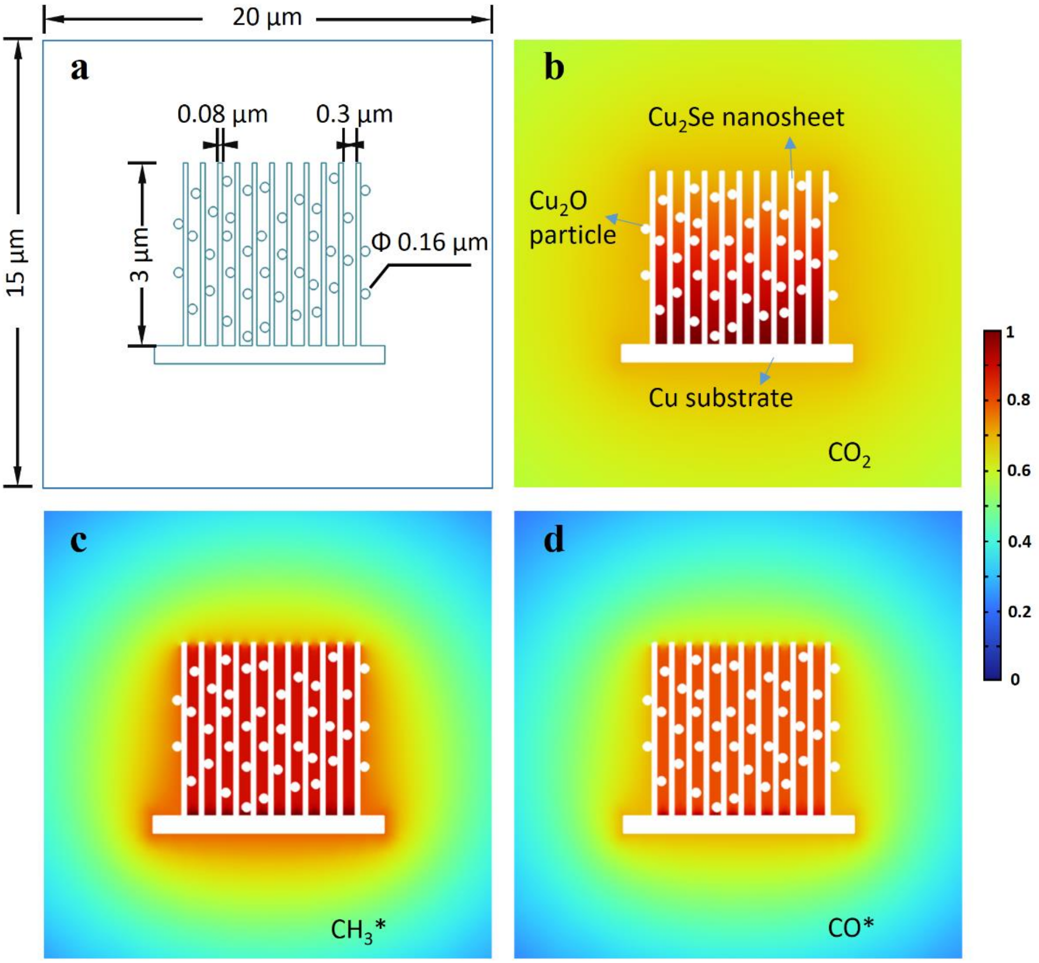

Supplementary Figure 48. Finite-element method simulations. (a) The size of the theoretical models. (b) Concentration distribution of CO_2. (c) Concentration distribution of CH_3* specie. (d) Concentration distribution of CO* specie.

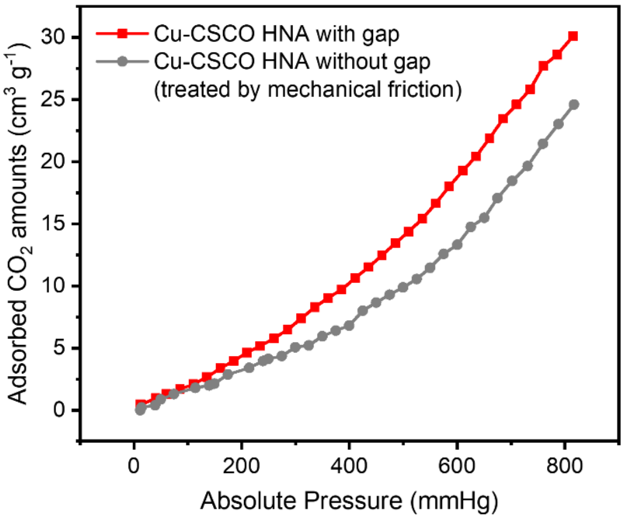

Supplementary Figure 49. CO_2 adsorption isotherms for Cu-CSCO HNA with (red) and without gap (black).

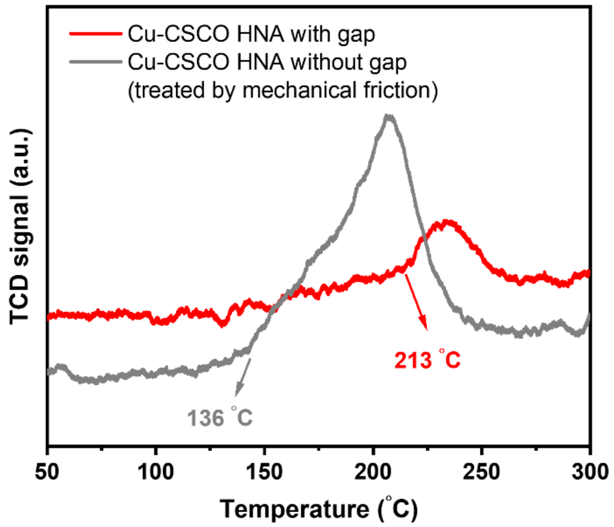

Supplementary Figure 50. CO-TPD measurement. The temperature of CO desorption for the Cu-CSCO HNA without gap (treated by mechanical friction method) is 136 ˚C, which is much smaller than that for the Cu-CSCO HNA with gap (213 ˚C), confirming the CO desorption is harder in the Cu-CSCO HNA system with gap.

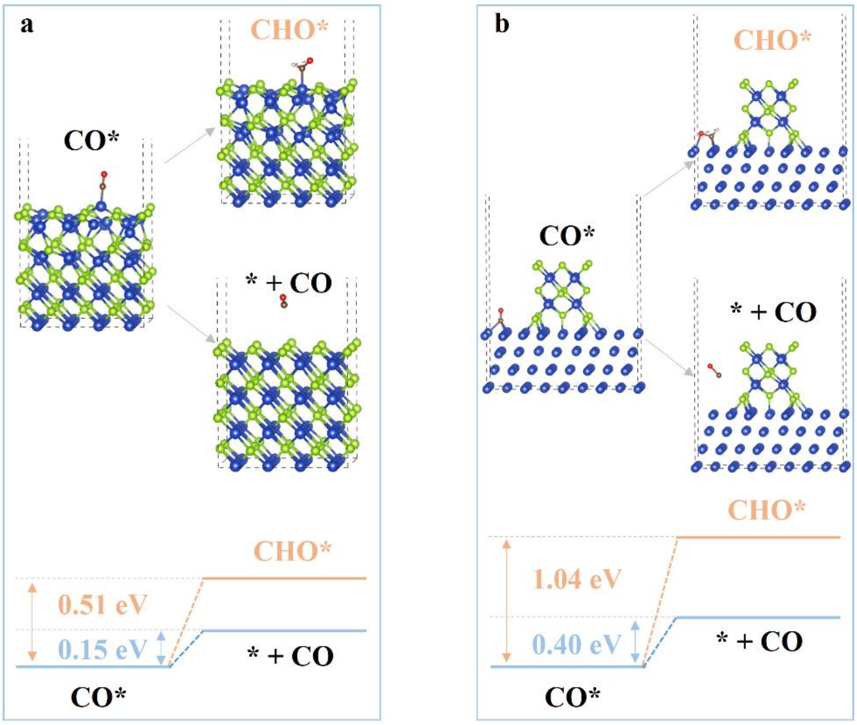

Supplementary Figure 51. The energy of CO desorption and protonation processes on the surface of (a) pure Cu_2Se and (b) Cu-Cu_2Se models.

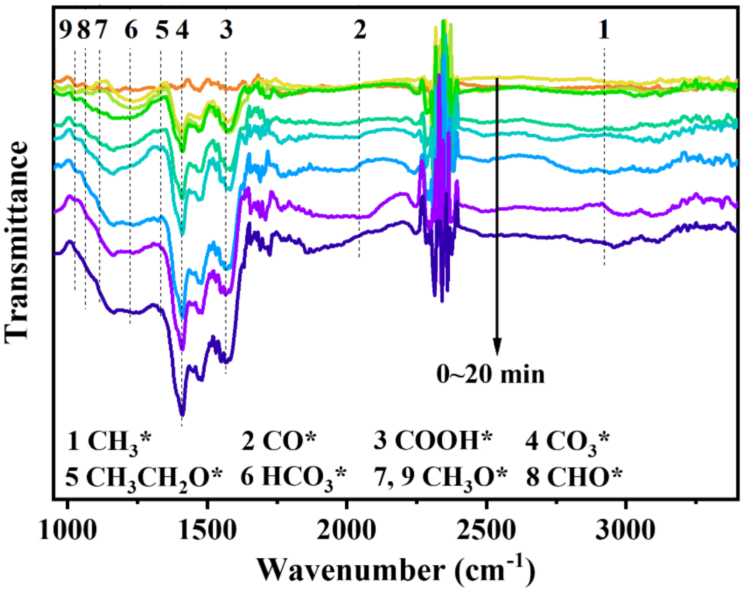

Supplementary Figure 52. ***In situ*** **FTIR spectroscopy characterization for co-adsorption of a mixture of CO_2 and H_2O vapor under light irradiation over CSCO heterojunction powder sprayed on Cu foil (CSCO-C).**

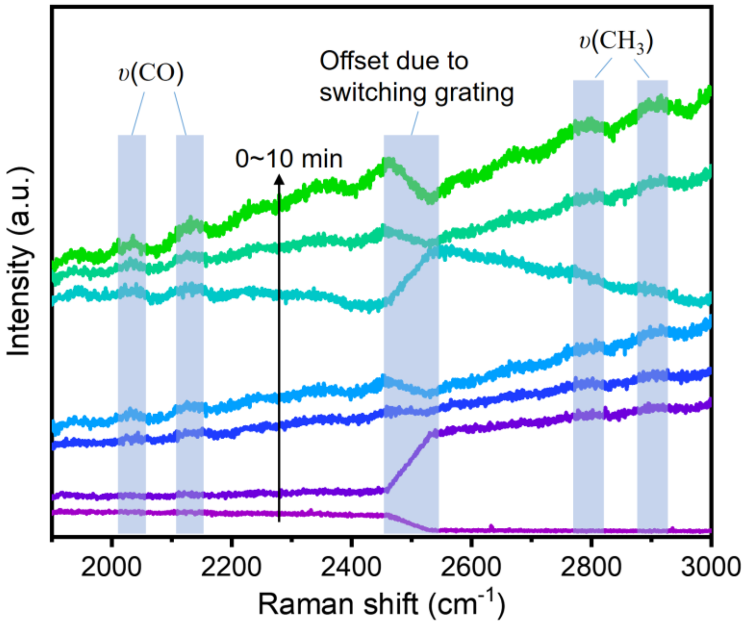

Supplementary Figure 53. ***In situ*** **Raman spectra characterization for co-adsorption of a mixture of CO_2 and H_2O vapor under light irradiation over Cu-CSCO HNA system.** The peaks at around 2040 and 2100 cm^{-1} are attributed to υ(CO) while the peaks at 2805 and 2924 cm^{-1} are assigned to the characteristic bands of $\upsilon(CH_3)$,[20-22] both of which are crucial initial intermediate for ethanol generation. The offset at 2500 cm^{-1} is due to switching grating.

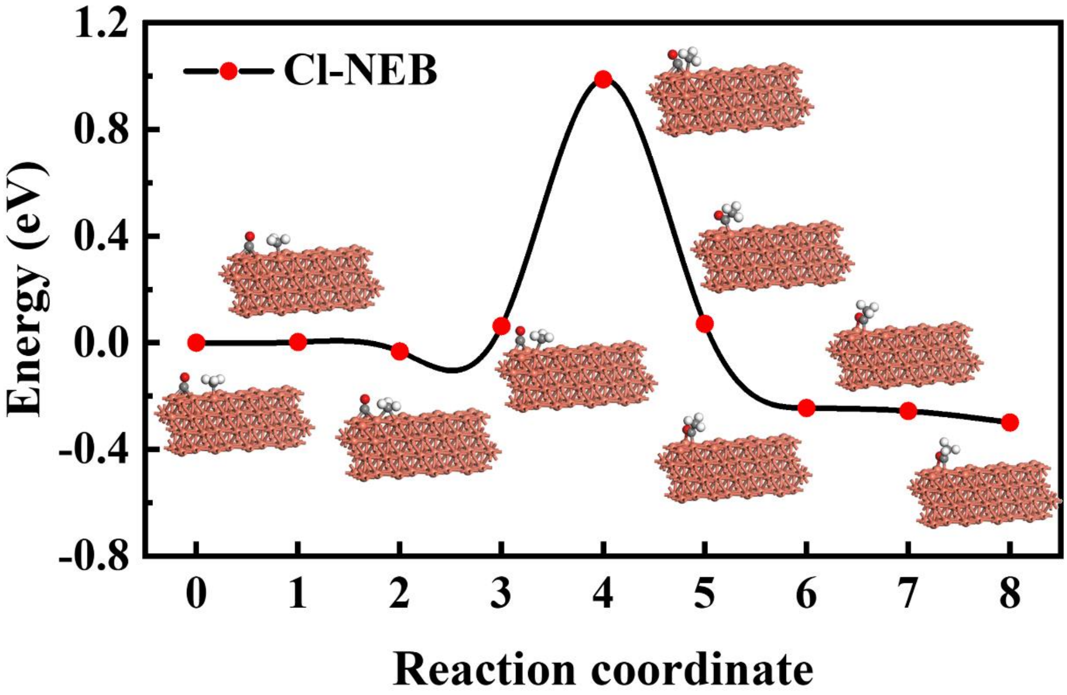

Supplementary Figure 54. Transition states (TS) of the coupling processes of CO* and CH_3* intermediates on the surface of pure Cu foil. The TS are calculated by the CI-NEB method, in which the energy barrier is computed to 0.99 eV. Brown ball: Cu; red ball: O; white ball: H; gray: C.

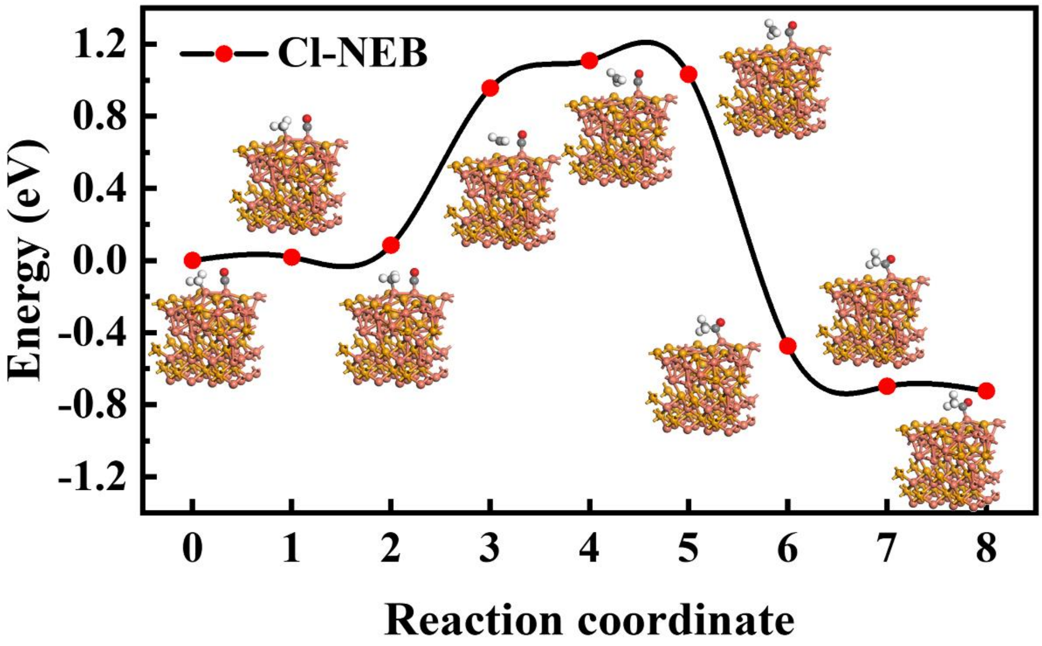

Supplementary Figure 55. TS of the coupling processes of CO* and CH_3* intermediates on the surface of pure Cu_2Se. The TS are calculated by the CI-NEB method, in which the energy barrier is computed to 1.12 eV. Brown ball: Cu; yellow: Se; red ball: O; white ball: H; gray: C.

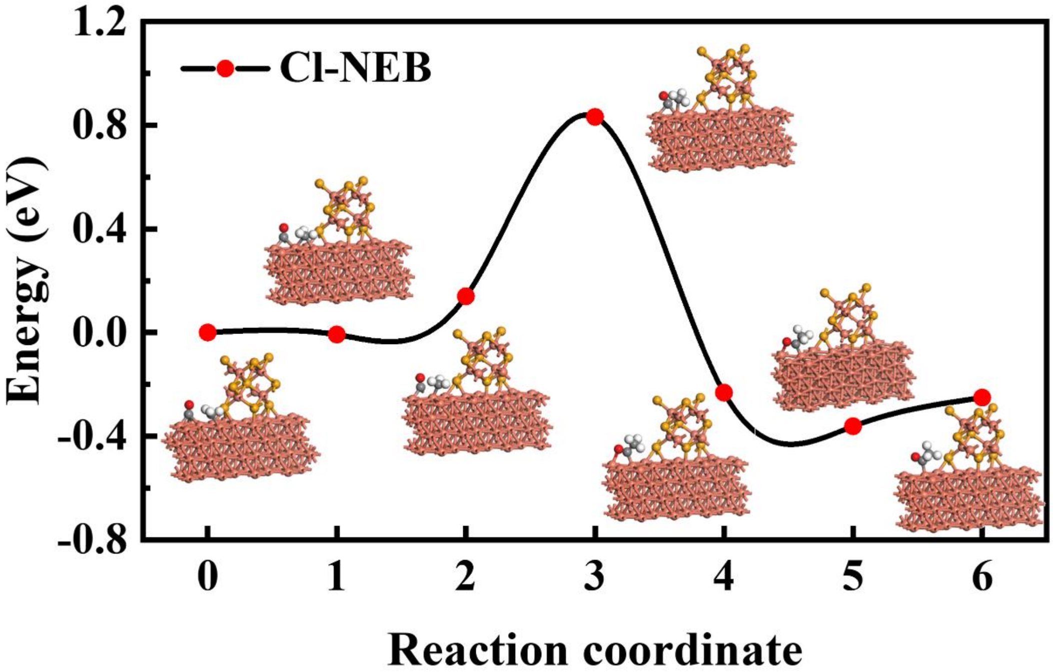

Supplementary Figure 56. TS of the coupling processes of CO* and CH_3* intermediates on the interface of Cu-Cu_2Se. The TS are calculated by the CI-NEB method, in which the energy barrier is computed to 0.83 eV. Brown ball: Cu; yellow: Se; red ball: O; white ball: H; gray: C.

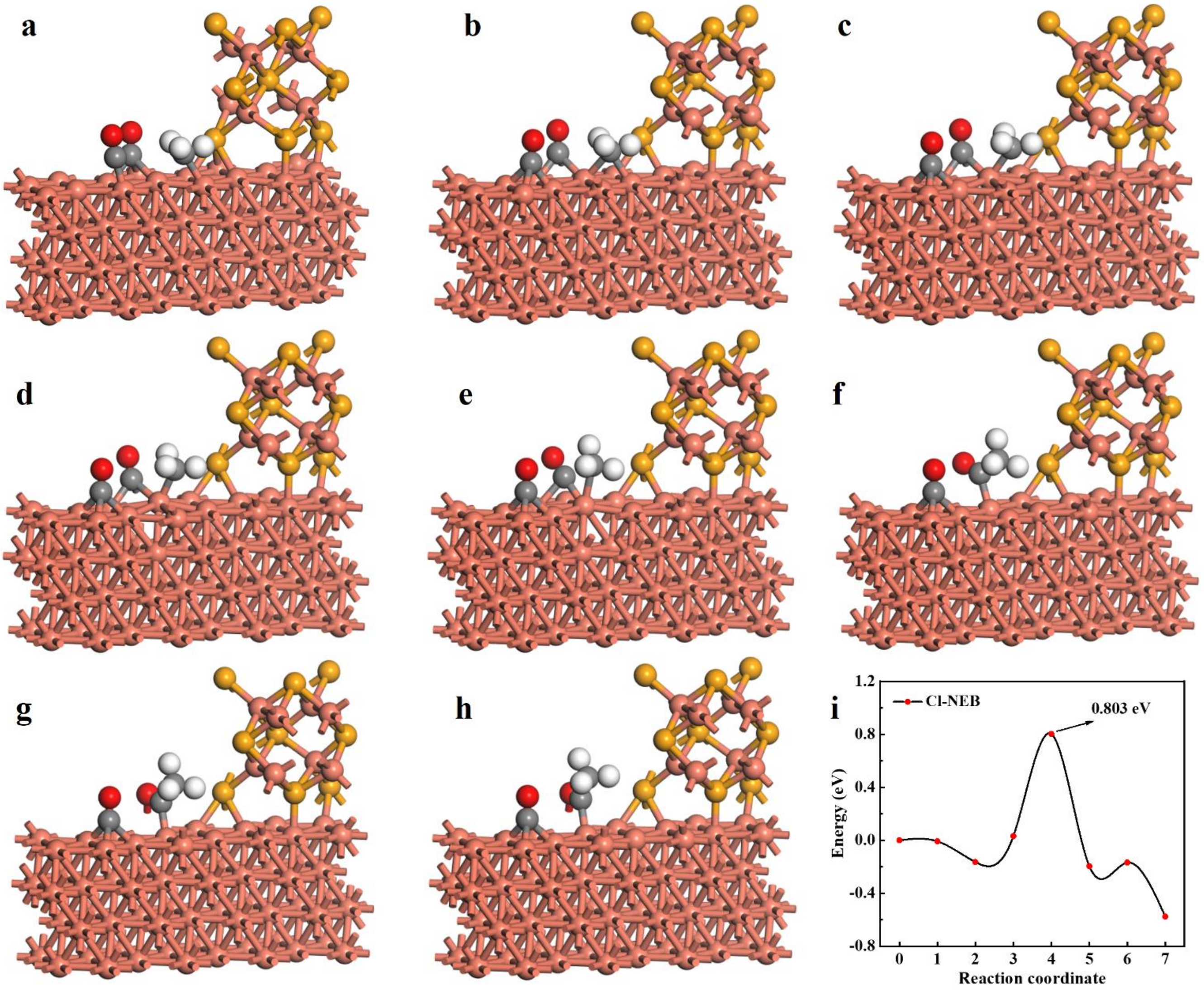

Supplementary Figure 57. TS of the coupling processes of CO* and CH_3* intermediates on the interface of Cu-Cu_2Se with 1 additional CO*. (a)-(h) The TS models. (i) The calculated TS energy plots. TS are calculated by the CI-NEB method, in which the energy barrier is computed to 0.803 eV. Brown ball: Cu; yellow: Se; red ball: O; white ball: H; gray: C.

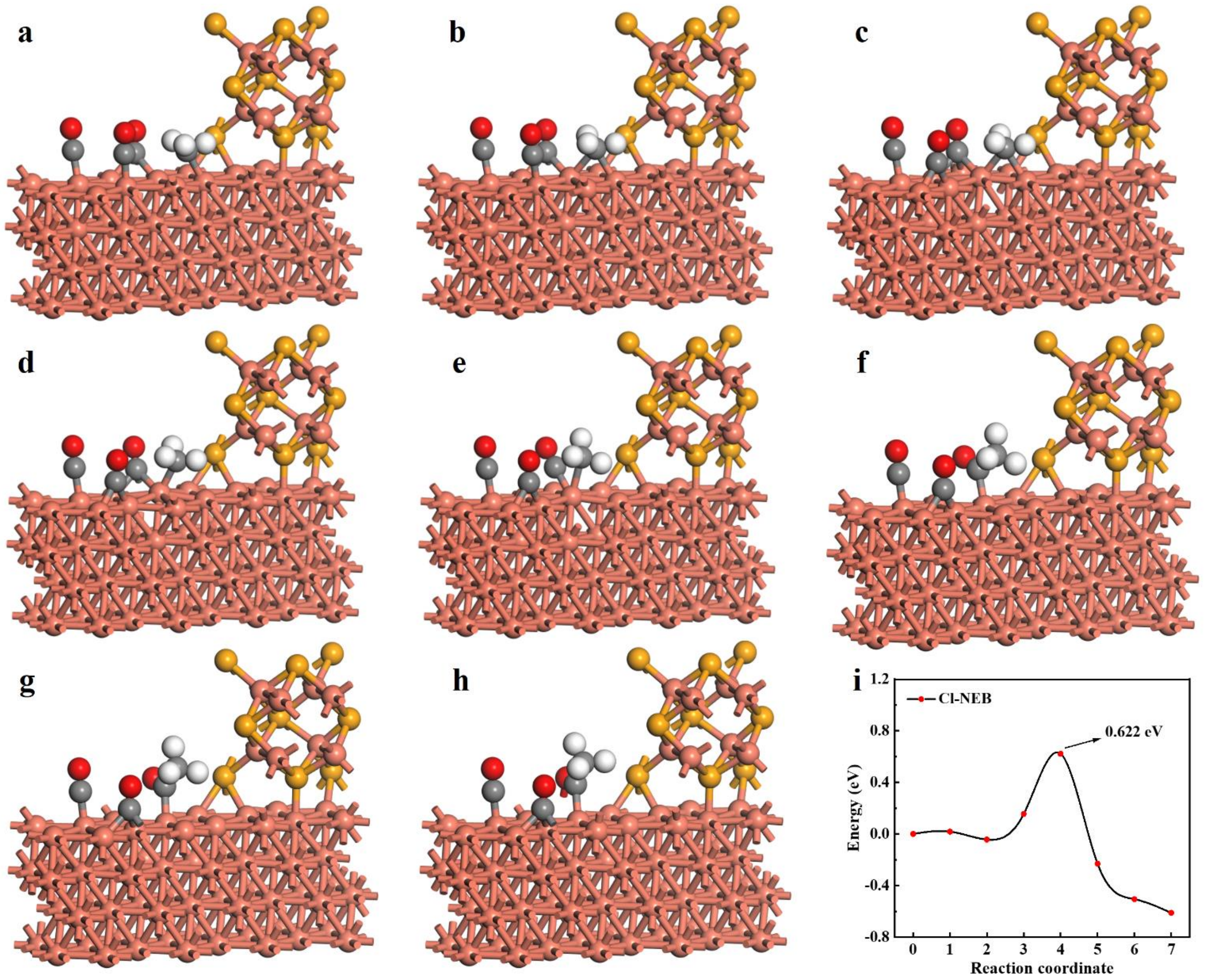

Supplementary Figure 58. TS of the coupling processes of CO* and CH_3* intermediates on the interface of Cu-Cu_2Se with 2 additional CO*. (a)-(h) The TS models. (i) The calculated TS energy plots. TS are calculated by the CI-NEB method, in which the energy barrier is computed to 0.622 eV. Brown ball: Cu; yellow: Se; red ball: O; white ball: H; gray: C.

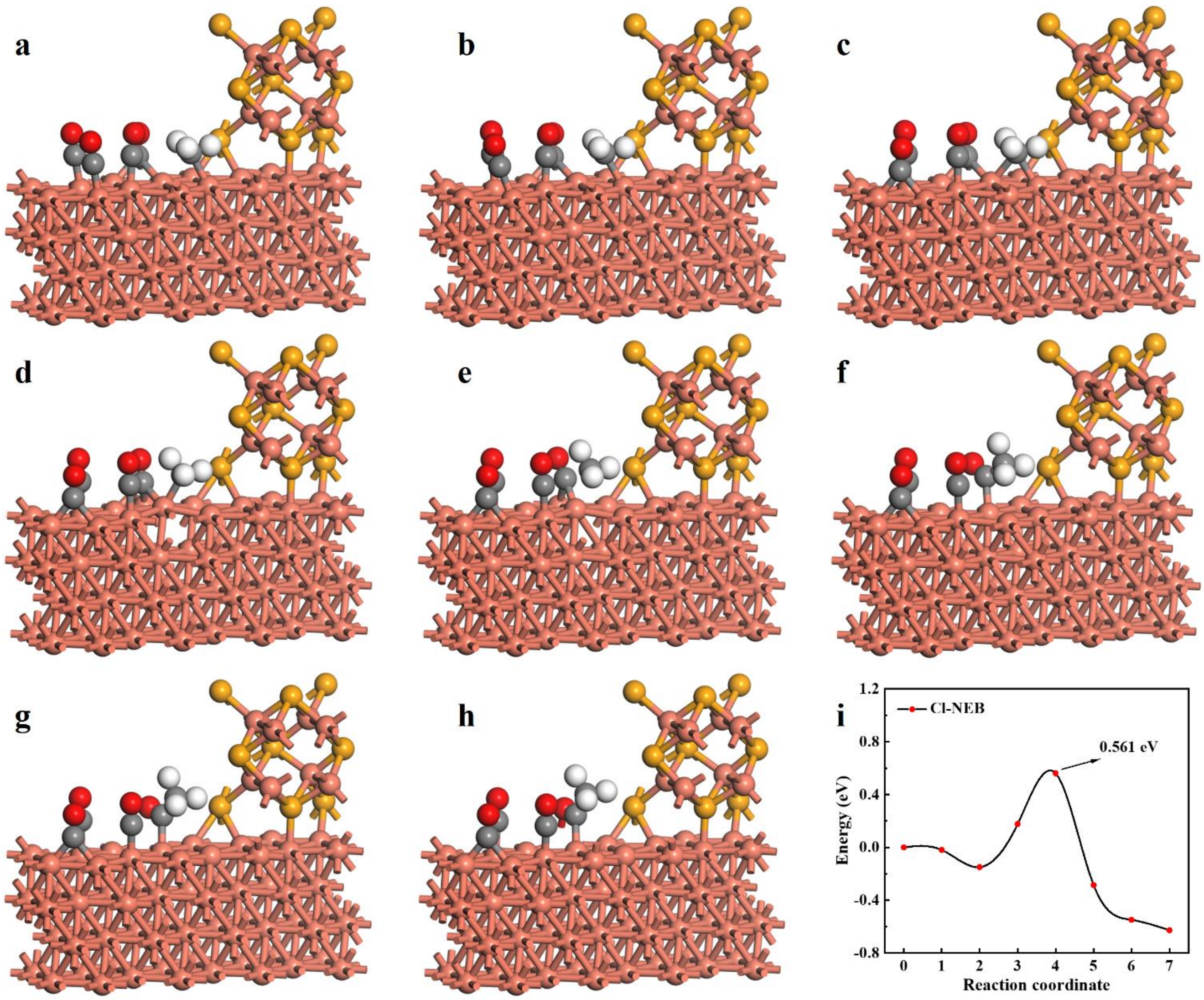

Supplementary Figure 59. TS of the coupling processes of CO* and CH_3* intermediates on the interface of Cu-Cu_2Se with 3 additional CO*. (a)-(h) The TS models. (i) The calculated TS energy plots. TS are calculated by the CI-NEB method, in which the energy barrier is computed to 0.561 eV. Brown ball: Cu; yellow: Se; red ball: O; white ball: H; gray: C.

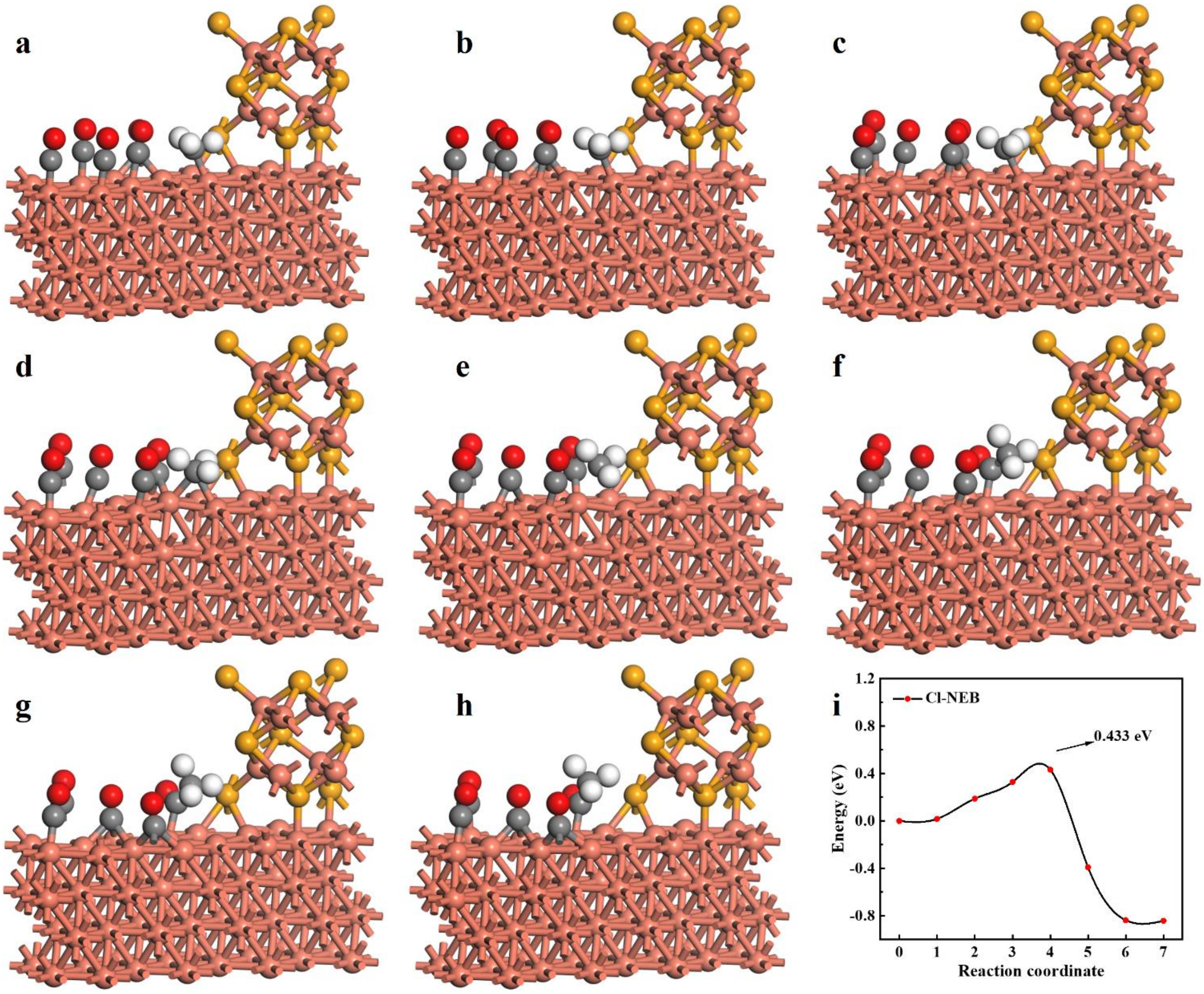

Supplementary Figure 60. TS of the coupling processes of CO* and CH_3* intermediates on the interface of Cu-Cu_2Se with 4 additional CO*. (a)-(h) The TS models. (i) The calculated TS energy plots. TS are calculated by the CI-NEB method, in which the energy barrier is computed to 0.433 eV. Brown ball: Cu; yellow: Se; red ball: O; white ball: H; gray: C.

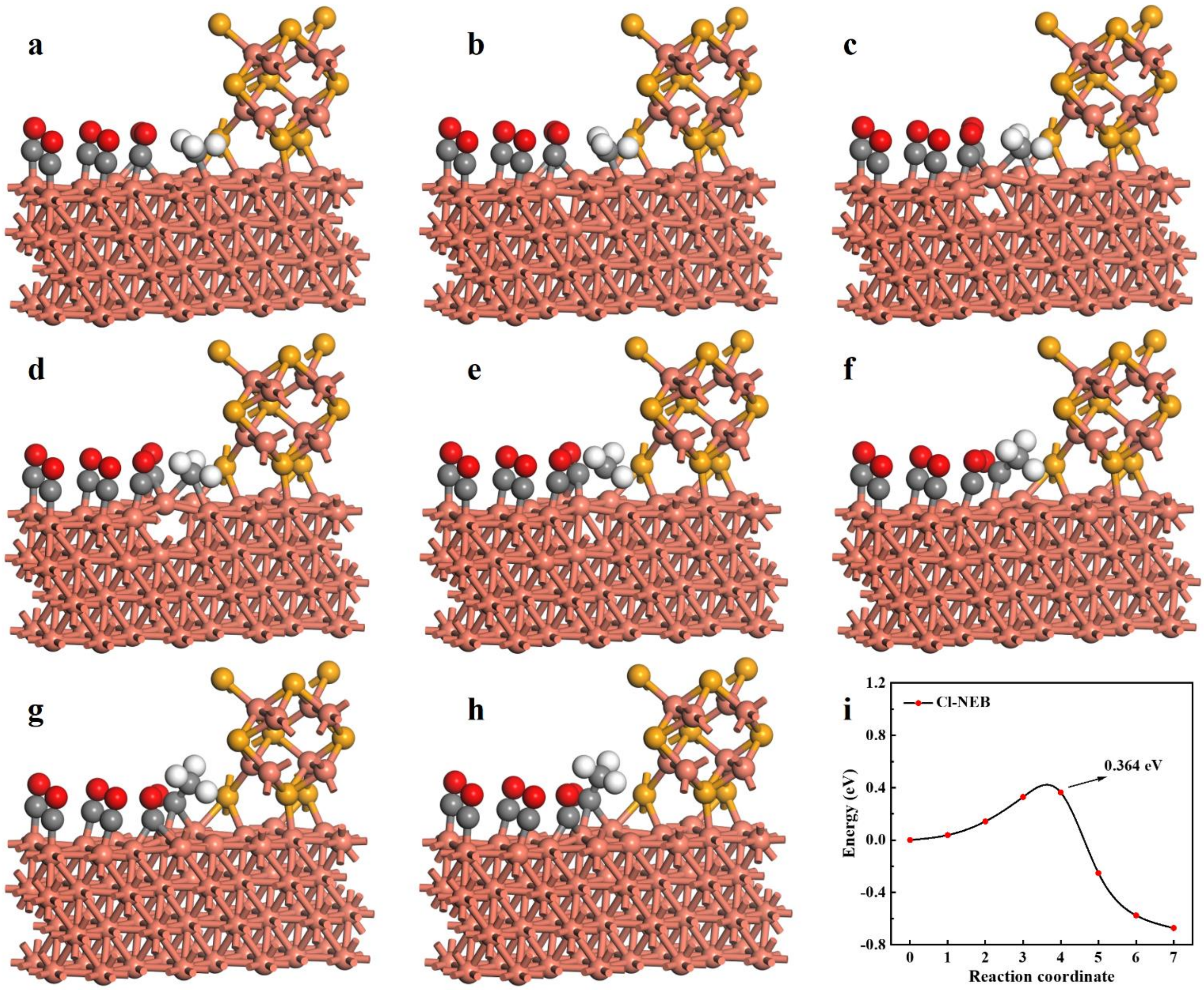

Supplementary Figure 61. TS of the coupling processes of CO* and CH_3* intermediates on the interface of Cu-Cu_2Se with 5 additional CO*. (a)-(h) The TS models. (i) The calculated TS energy plots. TS are calculated by the CI-NEB method, in which the energy barrier is computed to 0.364 eV. Brown ball: Cu; yellow: Se; red ball: O; white ball: H; gray: C.

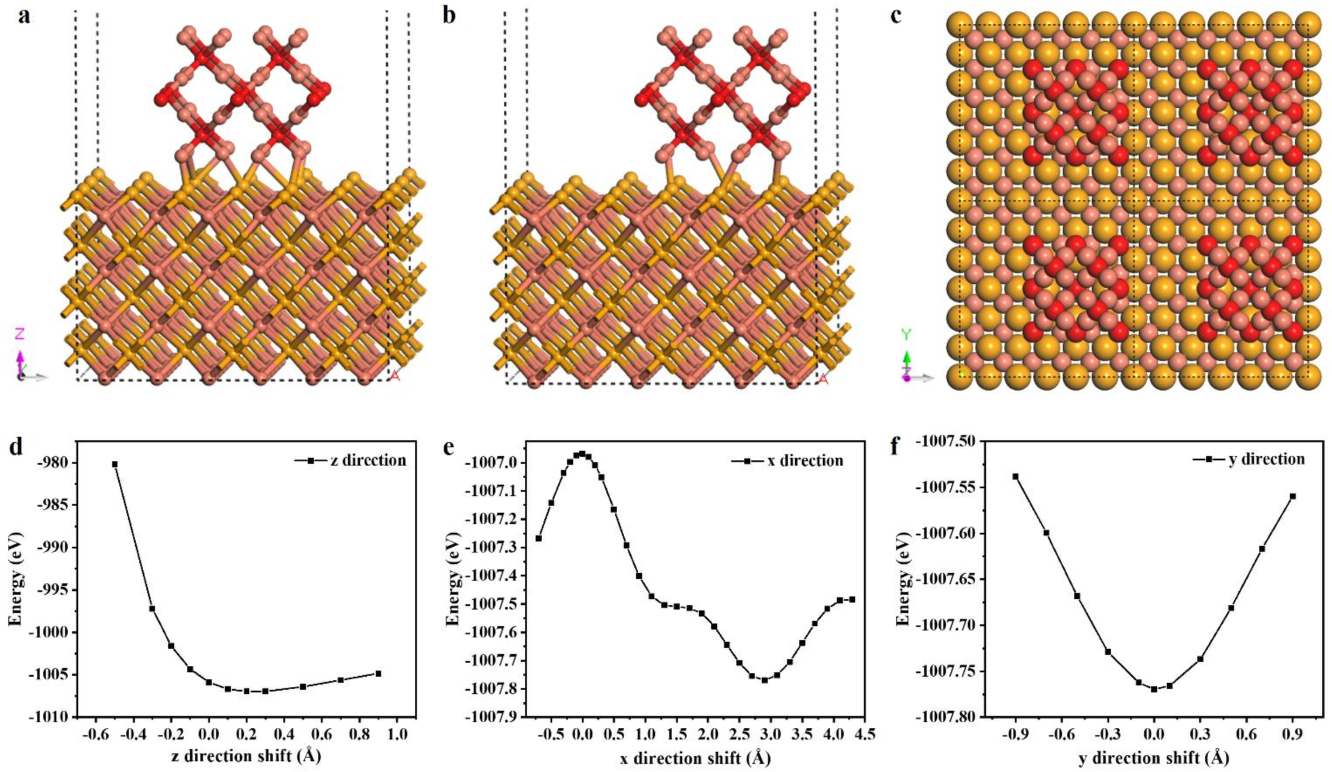

Supplementary Figure 62. The initial structure models and energy changes during the optimization processes. (a) Initial models of Cu_2Se-Cu_2O; (b)-(c) Views of the optimized initial models of Cu_2Se-Cu_2O along different directions; (d)-(f) total energy changes of the structure during optimization along z, x and y directions.

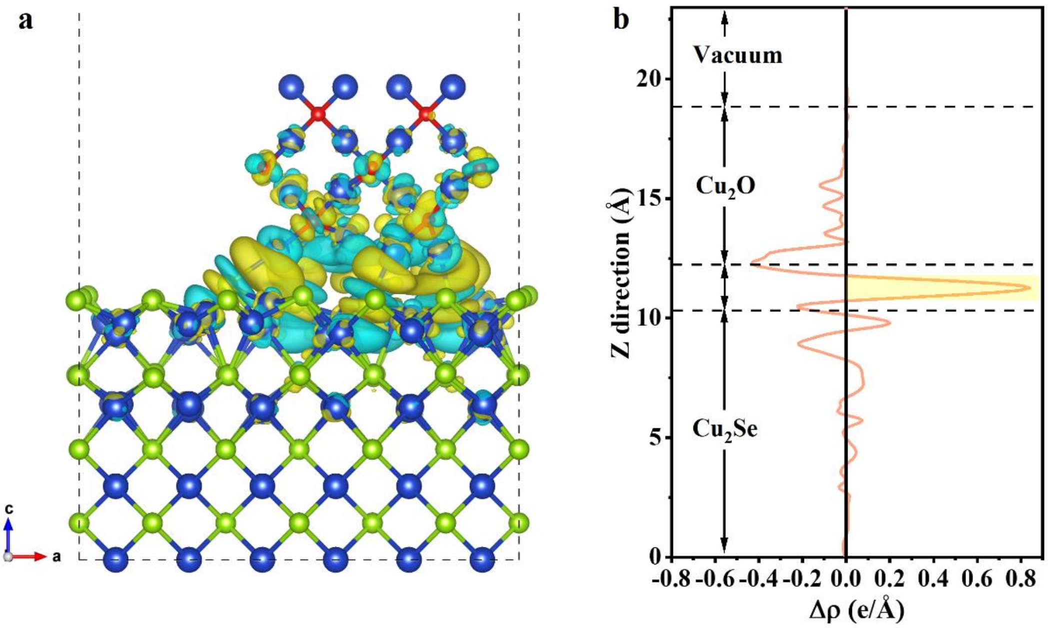

Supplementary Figure 63. CDD of CSCO heterostructure model. (a) Three dimensional distribution; (b) one dimensional distribution. The yellow and blue isosurfaces correspond to the increase in the number of electrons and the depletion zone, respectively. The isosurfaces are 0.001 e $Bohr^{-3}$. The enriched charge density at the interface is beneficial for H_2O adsorption.

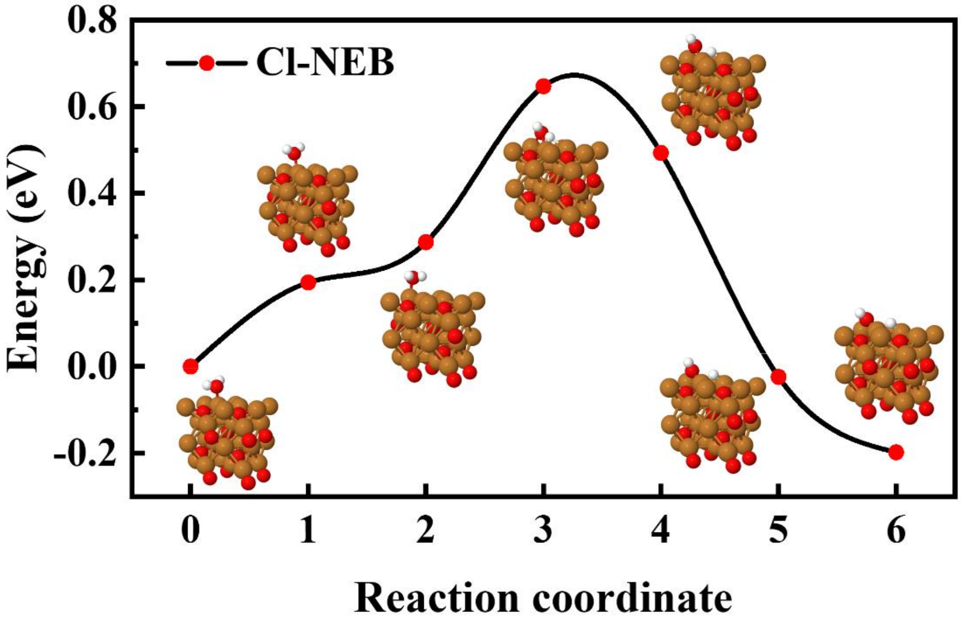

Supplementary Figure 64. Transition states (TS) of H_2O* dissociation into OH* and H* on the surface of pure Cu_2O. The TS are calculated by the climbing image nudged elastic band (CI-NEB) method, in which the energy barrier is computed to 0.647 eV. Brown ball: Cu; red ball: O; white ball: H.

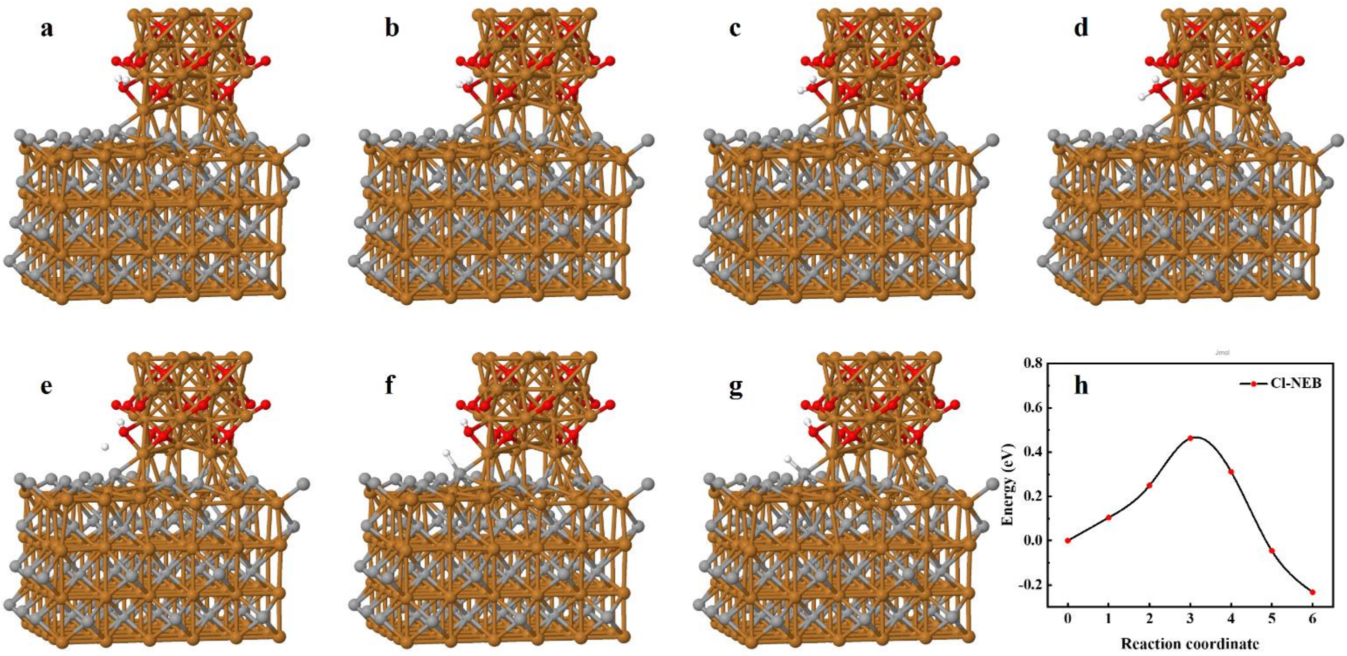

Supplementary Figure 65. Transition states (TS) of H_2O* dissociation into OH* and H* on the interface of CSCO. (a)-(g) The TS models. (h) The calculated TS energy plots. TS are calculated by the climbing image nudged elastic band (CI-NEB) method, in which the energy barrier is computed to 0.462 eV. Brown ball: Cu; gray ball: Se; red ball: O; white ball: H.

Supplementary Tables

Supplementary Table 1. Free energy correction for species during CO_2 conversion.

Unit (eV)	E	ZPE	TS
H_2	-6.76	0.27	0.40
CO_2	-22.98	0.31	0.66
H_2O	-14.21	0.56	0.67
CO	-14.79	0.13	0.6

Supplementary Table 2. Total energy and free energy correction for Cu_2Se slab and corresponding reaction intermediate models.

Unit (eV)	E	ZPE	TS
Cu_2Se (*)	-359.65	/	/
CO*	-375.12	0.18	0.12
CHO*	-378.25	0.45	0.20

Supplementary Table 3. Total energy and free energy correction for Cu-Cu_2Se slab and corresponding reaction intermediate models.

Unit (eV)	E	ZPE	TS
Cu-Cu_2Se (*)	-311.98	/	/
CO*	-327.66	0.17	0.15
CHO*	-330.34	0.42	0.13

Supplementary References

1. Wang, W. et al. Photocatalytic C-C coupling from carbon dioxide reduction on copper oxide with mixed-valence copper(I)/copper(II). *J. Am. Chem. Soc.* **143**, 2984-2993 (2021).
2. Kang, Q. et al. Photocatalytic reduction of carbon dioxide by hydrous hydrazine over Au-Cu alloy nanoparticles supported on $SrTiO_3/TiO_2$ coaxial nanotube arrays. *Angew. Chem. Int. Ed.* **54**, 841-845 (2015).
3. Chen, Q. et al. Photo-induced Au-Pd alloying at TiO_2 {101} facets enables robust CO_2 photocatalytic reduction into hydrocarbon fuels. *J. Mater. Chem. A.* **7**, 1334-1340 (2019).
4. Yu, L. et al. Enhanced activity and stability of carbon-decorated cuprous oxide mesoporous nanorods for CO_2 reduction in artificial photosynthesis. *ACS Catal.* **6**, 6444-6454 (2016).
5. Phongamwong, T. et al. Role of chlorophyll in Spirulina on photocatalytic activity of CO_2 reduction under visible light over modified N-doped TiO_2 photocatalysts. *Appl. Catal. B* **168-169**, 114-124 (2015).
6. Zheng, Y. et al. Triple-layered sandwich nanotube of carbon nanotube@TiO_2 nanocrystalline@carbon with superior lithium storage performance. *Mater. Res. Bull.* **133**, 111076 (2021).
7. Torres, J. et al. Experimental evidence of CO_2 photoreduction activity of SnO_2 nanoparticles. *ChemPhysChem.* **21**, 2392-2396 (2020).
8. Yu, S. et al. Plasmonic photosynthesis of C_1-C_3 hydrocarbons from carbon dioxide assisted by an ionic liquid. *Nat. Commun.* **10**, 2022 (2019).
9. Ni, B. et al. Correlating oxidation state and surface ligand motifs with the selectivity of CO_2 photoreduction to C_2 products. *Angew. Chem. Int. Ed.* **62**, e202215574 (2023).
10. Ma, M. et al. Ultrahigh surface density of Co-N_2C single-atom-sites for boosting photocatalytic CO_2 reduction to methanol. *Appl. Catal. B* **300**, 120695 (2022).
11. Wang, T. et al. Engineering catalytic interfaces in $Cu^{\delta+}/CeO_2$-TiO_2 photocatalysts for synergistically boosting CO_2 reduction to ethylene. *ACS Nano* **16**, 2306-2318 (2022).
12. Wu, Y. et al. Selective CO_2-to-C_2H_4 photoconversion enabled by oxygen-mediated triatomic sites in partially oxidized bimetallic sulfide. *Angew. Chem. Int. Ed.* **62**, e202301075 (2023).
13. Tahir, M. et al. Indium-doped TiO_2 nanoparticles for photocatalytic CO_2 reduction with H_2O vapors to CH_4. *Appl. Catal. B* **162**, 98-109 (2015).
14. Tian, F. et al. Visible-light-driven CO_2 reduction to ethylene on CdS: Enabled by structural relaxation-induced intermediate dimerization and enhanced by ZIF-8 coating. *Appl. Catal. B* **285**, 119834 (2021).
15. Gao, W. et al. Anchoring of black phosphorus quantum dots onto WO_3 nanowires to boost photocatalytic CO_2 conversion into solar fuels. *Chem. Commun.* **56**, 7777-7780 (2020).
16. Kou, M. et al. Photocatalytic CO_2 conversion over single-atom MoN_2 sites of covalent organic framework. *Appl. Catal. B* **291**, 120146 (2021).
17. Nguyen, T.-V. et al. Photoreduction of CO_2 over Ruthenium dye-sensitized TiO_2-based catalysts under concentrated natural sunlight. *Catal. Commun.* **9**, 2073-2076 (2008).
18. Liu, F. et al. Direct Z-scheme hetero-phase junction of black/red phosphorus for photocatalytic water splitting. *Angew. Chem. Int. Ed.* **58**, 11791-11795 (2019).
19. Zhao, D. et al. Boron-doped nitrogen-deficient carbon nitride-based Z-scheme heterostructures for photocatalytic overall water splitting. *Nat. Energy* **6**, 388-397 (2021).

20. Shan, J. et al. New aspects of operando Raman spectroscopy applied to electrochemical CO_2 reduction on Cu foams. *J. Chem. Phys.* **150**, 041718 (2019).
21. Yu, Y. Complete raman spectral assignment of methanol in the C-H stretching region. *J. Phys. Chem. A* **117**, 4377-4384 (2013).
22. Dieter, K. et al. Development of a Raman spectrometer for the characterization of gaseous hydrocarbons at high temperatures. *J. Quant. Spectrosc. Ra.* **277**, 107978 (2022).
