# Supplementary material for: Photothermal CO2 conversion to ethanol through photothermal heterojunction-nanosheet arrays
Source: Nat Commun. 2024 Jul 5;15:5639. doi: 10.1038/s41467-024-49928-0 (PMC11224241; doi:10.1038/s41467-024-49928-0)
Supplement: Supplementary file 3 — Description of Additional Supplementary Files [file 41467_2024_49928_MOESM3_ESM.pdf]

Description of Additional Supplementary Files

File Name: Supplementary Data 1
Description: This supplementary dataset includes all the information of the optimized structures.
